# Evaluating Social and Ecological Vulnerability of Coral Reef Fisheries to Climate Change

**DOI:** 10.1371/journal.pone.0074321

**Published:** 2013-09-11

**Authors:** Joshua E. Cinner, Cindy Huchery, Emily S. Darling, Austin T. Humphries, Nicholas A. J. Graham, Christina C. Hicks, Nadine Marshall, Tim R. McClanahan

**Affiliations:** 1 Australian Research Council Centre of Excellence for Coral Reef Studies, James Cook University, Townsville, Queensland, Australia; 2 Earth to Ocean Research Group, Simon Fraser University, Burnaby, British Columbia, Canada; 3 Coastal Research Group, Rhodes University, Grahamstown, South Africa; 4 Coral Reef Conservation Project, Wildlife Conservation Society, Mombasa, Kenya; 5 Ecosystem Sciences, Commonwealth Scientific and Industrial Research Organisation, Townsville, Queensland, Australia; 6 Marine Programs, Wildlife Conservation Society, Bronx, New York, United States of America; University of Gothenburg, Sweden

## Abstract

There is an increasing need to evaluate the links between the social and ecological dimensions of human vulnerability to climate change. We use an empirical case study of 12 coastal communities and associated coral reefs in Kenya to assess and compare five key ecological and social components of the vulnerability of coastal social-ecological systems to temperature induced coral mortality [specifically: 1) environmental exposure; 2) ecological sensitivity; 3) ecological recovery potential; 4) social sensitivity; and 5) social adaptive capacity]. We examined whether ecological components of vulnerability varied between government operated no-take marine reserves, community-based reserves, and openly fished areas. Overall, fished sites were marginally more vulnerable than community-based and government marine reserves. Social sensitivity was indicated by the occupational composition of each community, including the importance of fishing relative to other occupations, as well as the susceptibility of different fishing gears to the effects of coral bleaching on target fish species. Key components of social adaptive capacity varied considerably between the communities. Together, these results show that different communities have relative strengths and weaknesses in terms of social-ecological vulnerability to climate change.

## Introduction

Millions of the world’s poorest people depend on the ecosystem goods and services provided by coral reefs [1]. Coral reefs are particularly important for fishing and tourism, but also contribute to coastal protection and in some places have significant cultural values. Coral reefs are one of the most productive and biologically diverse aquatic environments on Earth, yet they are also one of the most ecologically sensitive to climatic change [2], [3], are currently undergoing large-scale changes [4], [5]. Consequently, evaluating the links between the social and ecological and dimensions of vulnerability to climate change is a priority for reducing difficult-to-reverse impacts on coral reefs and increasing human food security [6], [7].

Climate change is affecting coral reefs through alterations in the long-term mean environmental conditions, inter-annual cycles, and seasonality, and the frequency of extreme climate events [8]. The increasing frequency of extreme climatic events can affect fish habitat, productivity, and distribution, as well as impact directly on fishing operations and the physical infrastructure of coastal communities [9]. Extreme events such as high-intensity cyclones and increased sea surface temperatures can have profound impacts on coral reef ecosystems and the communities that depend on them [10], [11]. For example, elevated sea temperature events can cause corals to bleach and die. This can alter the goods and services that coral reefs provide by changing the species compositions of fish and potentially reducing reef fisheries productivity, and consequently harming reef-dependent people [6], [12], [13], [14], [15]. The current era of rapid anthropogenic-driven climate change has the potential to undermine coral-reef associated livelihoods [7].

An increasingly critical aspect of sustaining coral reefs and the livelihoods of dependent people is understanding the vulnerability of particular reefs and their associated human communities to climate change impacts [16], [17]. Vulnerability, in the context of social and environmental change, is defined as the state of susceptibility to harm from perturbations [18]. Understanding vulnerability in coral reef fisheries is complicated because there is considerable heterogeneity in: 1) places that experience climate change-related events such as coral bleaching; 2) the ways that coral reef ecosystems are affected by and can recover from these impacts; 3) the ways that societies and individuals are impacted by these changes; and 4) the capacity of people to cope with and adapt to these changes. Knowledge about how vulnerable a system is, and the specific conditions that make it vulnerable, can help to provide a foundation for developing key actions that minimize the impacts of environmental change on people.

The conceptual framework of vulnerability to climate change provides a basis for operationalizing and assessing the vulnerability of linked social and ecological systems [19], [20]. A framework promoted by the Intergovernmental Panel on Climate Change (IPCC) [8] has been widely adopted for vulnerability assessments [21] (Figure S7 in File S1). The framework suggests that the extent to which people’s livelihoods are vulnerable to the impacts of climate change is dependent on: 1) their exposure to climate impacts (i.e. if impacts are felt in their location); 2) their sensitivity (i.e. the extent to which their livelihood is affected by an impact); and 3) their capacity to adapt to the likely impacts [16], [18], [19], [20], [22], [23], [24].

Exposure is the degree to which a system is stressed by climate, such as the magnitude, frequency, and duration of a climatic event such as temperature anomalies or extreme weather events [18], [25]. In a practical sense, exposure is the extent to which a region, resource, or community experiences change [8]. For fishing communities, exposure captures how much the resource they depend on will be affected by environmental change. In tropical reef fisheries, exposure can vary depending on factors such as oceanographic conditions, prevailing winds, and latitude, which can increase the likelihood of being impacted by events such as cyclones or coral bleaching [26]. Sensitivity, in the context of environmental change, is the susceptibility of a defined component of the system to harm, resulting from exposure to stresses [18]. The sensitivity of social systems depends on economic, political, cultural and institutional factors that allow buffering or attenuation of change. For example, social systems are more likely to be sensitive to climate change if they are highly dependent on a climate-vulnerable natural resource. Sensitivity can confound (or ameliorate) the social and economic effects of climate exposure. Adaptive capacity is a latent characteristic that reflects peoples’ ability to anticipate and respond to changes, and to minimize, cope with, and recover from the consequences of change [22]. For example, people with low adaptive capacity may have difficulty adapting to change or taking advantage of the opportunities created by changes in the availability of ecosystem goods and services stimulated by climate change or changes in management.

The above examples illustrate the three dimensions of social vulnerability, but they also have ecological components. For example, the sensitivity of ecological systems to climate change can include physiological tolerances to change and/or variability in physical and chemical conditions (i.e. temperature, pH, etc.), such as certain corals that are highly sensitive to increases in sea temperatures. This creates the need to evaluate both systems and, therefore, a new multi-disciplinary literature on the vulnerability of linked social-ecological systems to climate change has emerged [16], [23], [27], [28]. The central idea behind linked or coupled social-ecological systems is that human actions and social structures profoundly influence ecological dynamics, and vice-versa [18], [29].

### Modified Vulnerability Framework

**Figure 1:**
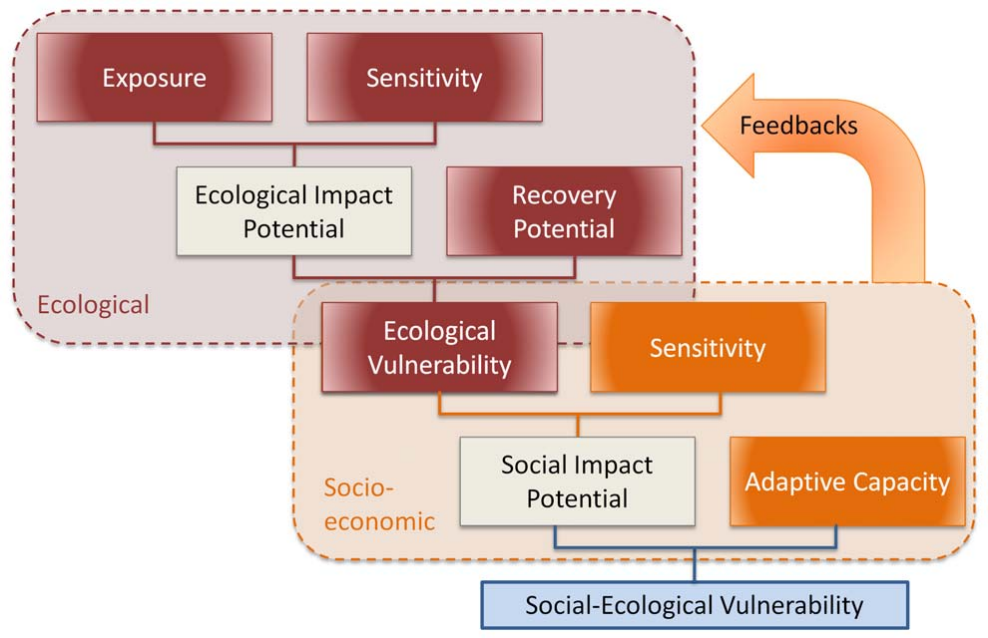
Heuristic framework for linked social-ecological vulnerability. In the ecological domain, ecological exposure and ecological sensitivity create impact potential. The impact potential and the ecological recovery potential together form the ecological vulnerability, or exposure in the social domain. This ecological vulnerability combined with the sensitivity of people form the impact potential for society. The social adaptive capacity and the impact potential together create social-ecological vulnerability. Adapted from Marshall et al. [40], [53].

Here, we use a case study from the Kenyan coral reef fishery to operationalize a modification of the IPCC vulnerability framework. Our aim is to improve on previously developed applications of vulnerability in fisheries [e.g. 30,31] by explicitly considering both social and ecological dimensions of vulnerability. Our specific modification to the IPCC framework entails linking two vulnerability sub-models: one represents the components of ecological vulnerability to exposure to climate change, while the other represents social vulnerability to changes in the ecological system (Figure 1). In our modified framework, the potential impact of climate change on ecological systems results from the physical exposure to climatic stressors combined with the sensitivity of those ecosystems. Whether these potential impacts are fully experienced in the long term depends on the potential of the ecosystem to recover its basic structure and function in response to impacts. Thus, the combination of ecological exposure, ecological sensitivity, and ecological recovery potential (together what we refer to as ecological vulnerability) result in the degree to which climate change will impact on the continued supply of ecosystem goods and services. In turn, this ecological vulnerability represents the exposure of the socioeconomic domain to climate threats. The overall social-ecological vulnerability is then a result of the sensitivity of socioeconomic systems to ecological vulnerability, and the adaptive capacity of the society to adapt to such impacts (Figure 1).

### The Social and Ecological Context of the Kenyan Case Study

Our Kenyan case study demonstrates how assessments of exposure, sensitivity, and adaptive capacity can be undertaken for both social and ecological subsystems and provide an overall assessment of system vulnerability. Kenya presents an interesting case study to evaluate social-ecological vulnerability for four key reasons. First, in a comparison of vulnerability across five Western Indian Ocean countries, Kenyan sites are the most vulnerable overall [30], but there is considerable spread in both sensitivity and adaptive capacity. Indeed, much of the variability encountered in the region is contained within Kenya. Second, Kenya is at the frontline of climate change- its reefs were severely impacted by the 1998 El Nino-related coral-bleaching event. Temperature records suggest that the scale of this temperature anomaly was unprecedented [32], [33] and resulted in high levels of coral mortality in the northern Indian Ocean [34]. Consequently, extreme climate events are a current reality rather than a distant possibility. Third, people in coastal Kenya are heavily dependent on fisheries and other natural resources for their livelihoods [35]. Fishing in Kenya is typically conducted from the beach to the fringing reef within the sand, coral, and seagrass habitats of the fringing reef lagoon. Last, Kenya has a range of marine resource governance regimes, ranging from large national marine parks enforced by paramilitary organizations to largely open access areas where regular use of destructive beach seine nets damage marine habitats. In between are community controlled co-managed areas called Beach Management Units (BMUs) [36], [37]. In recent years, BMUs have started developing community-based fishery closures. Together this governance spectrum presents an opportunity to examine whether, and how different, governance regimes have the potential to influence vulnerability.

## Materials and Methods

### Ecological Sampling

**Figure 2:**
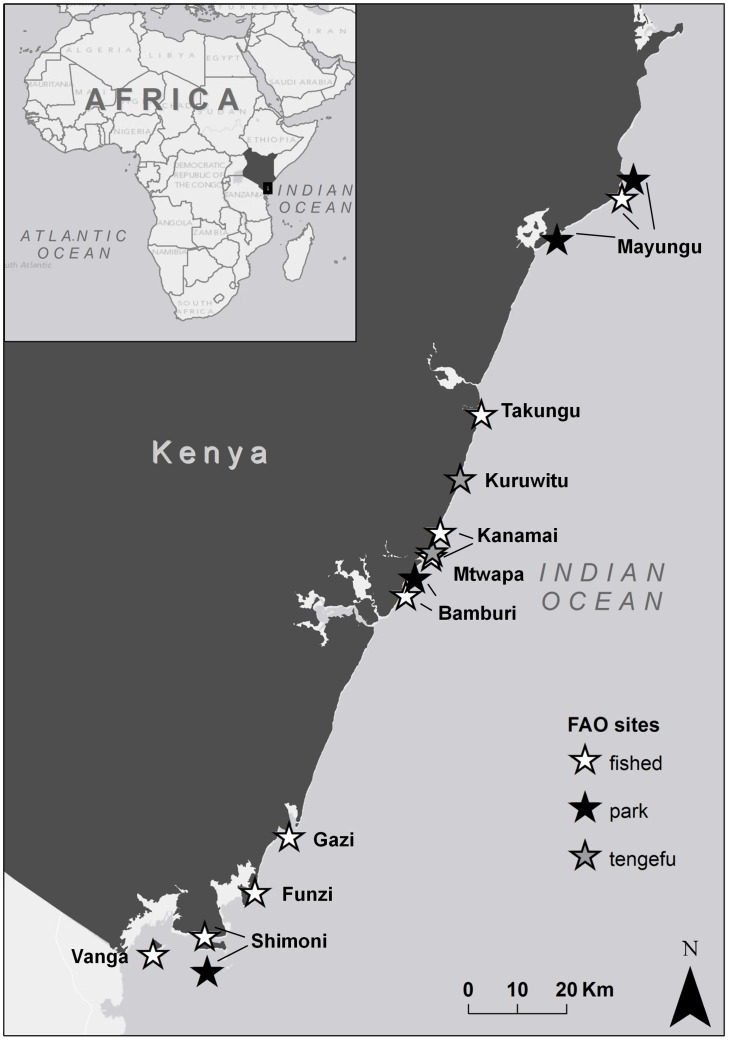
Map of study sites.

In 2009, 2011, and 2012 we surveyed 15 ecological sites associated with 10 coastal communities along the Kenyan coast, including heavily fished reefs, reefs within small, recently established community co-managed fisheries closures (“tengefus” in Swahili), and reefs in larger, well established no-take National Marine Parks managed by the Kenya Wildlife Service (Figure 2). All reef surveys were conducted in shallow back-reef flat habitat or shallow reef slope (<4 m). This depth was chosen partly because the extensive shallow back reef habitats along the Kenyan coast make up the majority of the available reef habitat. More importantly though, this back-reef lagoonal system is where the majority of the reef fishery activities are concentrated, meaning our ecological and social data are tightly coupled. Surveys were conducted in 2011 and 2012, with the exception of the Kisite Marine National Park, which was surveyed in 2009 (marked as Shimoni Park in Figure 2). At each site, we used standard underwater survey methods to evaluate coral reef benthic habitat and associated reef fish communities (Methods S1 in File S1).

### Ecological Indicators of Vulnerability

We developed metrics to explain key aspects of the ecological exposure, ecological sensitivity, and recovery potential of coral reef ecosystems to the impacts of climate change-associated coral bleaching (Table 1):

**Table 1:** Indicators of ecological sensitivity and ecological recovery potential.

		Statement of evidence	Weight of scientific evidence (−5 to 5)
**Ecological sensitivity indicators**			
**Coral**			
Coral bleaching susceptibility		Some species (e.g. branching or plating corals) are often severely impacted by disturbance and ahigh abundance of these species confers higher sensitivity	4.07
**Fish**			
Fish bleaching susceptibility		Certain fish species are more heavily impacted by disturbance and a high abundance of thesespecies confers higher sensitivity	3.2
**Recovery potential indicators**			
**Autotrophs/Corals**			
Coral cover	Coral cover is linked to increased resilience and recovery but most field studies showing no correlationbetween coral cover pre- or post-disturbance with recovery rates.		2.27
Coral to macroalgae cover	Macroalgae is a significant factor limiting the recovery of corals following disturbance by increasingcompetition for benthic substrate, allelopathy and by trapping sediment that smothers coral recruits.		3.37
Calcifying to non-calcifying cover	Calcifying organisms are important for reef framework (e.g., processes of settlement, recruitment andcementation of reef structure) and more calcifying organisms relative to non-calcifying organisms areexpected to increase or accelerate recovery following disturbances. However, the interactiveeffects of settlement induction, competition and increased predation makethe influence unclear.		1
Coral size distribution	There is scientific evidence that evenness across size classes increases recovery. An even distribution acrosssize classes indicates a recovering community of coral recruits, juveniles and adult colonies,whereas the under-representation of juvenile colonies suggests recruitment failure anda suppressed recovery rate. Moreover, the lack of large adult coral colonies may limitspawning stock and indicate environmental stress that has caused partial colonymortality and fragmentation.		2.5
Coral richness	Coral richness is expected to promote recovery, however there is limited evidence that coral diversitypromotes recovery following disturbance.		2.5
**Heterotroph/Fish**			
Fish biomass	Fish biomass in indicates the status of the fish stock, its potential growth, and is a proxy forecological metabolism		4.5
Herbivore grazing rate relativeto algal production	Most studies have linked increased herbivory to reduced macroalgal cover and an increase in coralrecruitment despite higher corallivory. One study has gone further and shown that increased herbivorebiomass led to a reversal in the reef trajectory from one of coral decline to coral recovery.Relative importance of fish and urchins varies geographically and with fishing intensity.		3.32
Fish species richness	Species richness is often used as a proxy for functional redundancy and is expected to promoteecological recovery by avoiding undesired ecological states.		3.5
Substrate complexity (rugosity)	Evidence that habitat complexity promotes recovery for corals occurs at small-scale sediment tilesbut has not been scaled up. There is good evidence that habitat complexity promotes refugeand recovery for fish		1.52
Fish size distribution	Large individuals in an assemblage indicate more even size-spectra and can increase fecundityto promote recovery of fish communities.		4
Herbivore functional diversity	Experimental evidence indicates that the presence of a diverse guild and functional groups of herbivores(reef fishes, sea urchins) can enhance coral recovery.		2.46

Weight of scientific evidence examines the consistency and type of evidence for each component, following the method of McClanahan et al. [39].

1. Ecological exposure to coral bleaching was described by a previously published multivariate model of how temperature, light, currents, tidal variation, chlorophyll, and water quality combine to create environmental conditions that make a site susceptible to coral bleaching impacts [26], [38]. Higher exposure values indicated environmental conditions that were more likely to result in thermal stress and subsequent coral bleaching, while lower values indicated sites that were less likely to experience thermal stress and coral bleaching (Methods S1 in File S1).
2. We estimated the ecological sensitivity of a site to coral bleaching using two indicators: i) the susceptibility of the coral community to bleaching; and ii) the susceptibility of the fish community to population declines associated with coral habitat loss from bleaching (Table 1, Methods S1 in File S1).
3. At each site, we collected information on ten ecological indicators of recovery potential (Table 1 **,** Methods S1 in File S1). These were: 1) hard coral cover; 2) the ratio of coral to macroalgae cover; 3) coral size distribution; 4) coral richness; 5) fish biomass; 6) fish species richness; 7) substrate complexity; 8) fish size distribution; 9) herbivore (fishes and sea urchins) diversity; and 10) an index of herbivore grazing relative to algal production. These indicators were weighted based on the scientific evidence that they contribute to recovery [39] (Table 1, Methods S1 in File S1).

We normalized each indicator between 0 and 1 (Methods S1 in File S1). Normalized indicators were averaged into composite metrics of sensitivity and recovery using an evidence-weighted framework based on expert opinion that evaluated the strength of evidence in support of each indicator [39] (Table 1). Ecological vulnerability was then estimated as [ecological exposure+ecological sensitivity] – Recovery Potential.

### Socioeconomic Data Collection

In 2010, we employed a combination of surveys targeted at resource users’ (fishermen, fish sellers, etc.) households and semi-structured interviews with key informants (community leaders, resource users, and other stakeholders) to gather information about their sensitivity and adaptive capacity to changes in the coral reef system. We triangulated results in each study site. In total, we conducted 310 household surveys, 9 key informant interviews, 10 community leader interviews, and 10 organizational leader interviews. All interviews were conducted in Swahili by trained interviewers. Respondents for the household surveys were randomly selected from lists of resource users provided by local leaders. Lists were cross-referenced with other fishermen for accuracy. We sampled 38% of approximately 810 resource users from our study sites. Key informant interviews were conducted using three semi-structured interview forms to specifically target: 1) knowledgeable fishers; 2) community leaders, and 3) fishery landing site leaders. Key informants were selected using non-probability sampling techniques.

### Social Indicators of Vulnerability

Based on all of these survey types, we generated 13 socioeconomic indicators, which we separated into social sensitivity and adaptive capacity measures (Table 2, Methods S1 in File S1). We developed a metric of sensitivity based on two key aspects: 1) the level of dependence on marine resources [31], [40]; and 2) data on how susceptible the catch composition of different gears were to climate change impacts [41], [42]. Information on these two aspects of sensitivity was combined into a single metric of social sensitivity (see Methods S1 in File S1 for detailed description). We modified the adaptive capacity index developed in McClanahan et al. [43] and Cinner et al. [30]. Based on both the household surveys and key informant interviews described above, we examined 11 social indicators of local-scale adaptive capacity (Table 2 and S5 in File S1). These were combined into a single, un-weighted metric of social adaptive capacity (Methods S1 in File S1).

**Table 2:** Indicators of social adaptive capacity.

Indicator	Description	Bounding
**Human agency** *“HumanAgency”*	Recognition of causal agents impacting marine resources (measured by content organizing responses to open-ended questions about what could impact thenumber of fish in the sea)	Binomial: 0; 1
**Access to credit** * *“AccessCredit”*	Measured as whether the respondent felt he or she could access credit throughformal institutions or informal means (e.g., family, friends, middlemen/dealers)	Binomial: 0; 1
**Occupational mobility** *“OccupMob”*	Indicated as whether the respondent changed jobs in the past five years andpreferred their current occupation	Binomial: 0; 1
**Occupational multiplicity** *“OccupMult”*	The total number of person-jobs in the household	Continuous: 1^st^ quartile = 1; 3^rd^ quartile = 3
**Social capital** *“SocialCapital”*	Measured as the total number of community groups the respondent belonged to	Continuous: min = 0; max = 3
**Material style of life** *“MSL”*	A material style of life indicator measured by factor analyzing whether respondentshad 15 material possessions such as vehicle, electricity and the type of walls,roof, and floor	Continuous: 1^st^ quartile; 3^rd^ quartile
**Gear diversity** *“GearDiv”*	Technology (measured as the diversity of fishing gears used); 8) infrastructure	Binomial: 0 = 1 gear; 1 = more than 1 gear
**Community infrastructure** *“CommInfrastr”*	Infrastructure	Continuous: min = 0; max = 26
**Trust** * *“Trust”*	Trust- measured as an average of Likert scale responses to questions abouthow much respondents trusted community members, local leaders, police,and local government	Continuous: min = 0.8; max = 5
**Capacity to change^2012^** *“CapacityChange”*	Capacity to anticipate change and to develop strategies to respond (measured bycontent organizing responses to open ended questions relating to a hypothetical50% decline in fish catch)	Binomial: 0; 1
**Debt** * **^2012^** *“NoDebt”*	Measured as whether or not the respondent was presently in debt of more than1 week’s salary (this indicator negatively contributed to adaptive capacityso we took the inverse).	Binomial: 0 = in debts; 1 = not in debts

2012 = only used for 2012 analysis.

* = New indicators added to the adaptive capacity compare to previous.

### Analysis

We compared the three aspects of ecological vulnerability (ecological exposure, ecological sensitivity, and ecological recovery potential) across the three management groups (fished reefs, tengefus, and no-take marine reserves) using a one-way analysis of variance. We described the multivariate relationships among the ecological exposure, ecological sensitivity, and ecological recovery potential indicators of ecological vulnerability using a correlation-based Principal Components Analysis (PCA) on Euclidean distances among indicators. We visualized the differences among the three components of ecological vulnerability using a bubble plot, where ecological sensitivity was plotted against ecological recovery potential and ecological exposure was indicated by the size of the points. These three components were combined into a metric of ecological vulnerability, which was then used as the exposure component of the social-ecological vulnerability (Figure 1, equation 1) as follows:
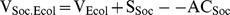
Where V is vulnerability, E is exposure, S is sensitivity, AC is adaptive capacity, _Soc_ is social, _Ecol_ is ecological, and V_Ecol_ = E_Ecol_+S_Ecol_ – Recovery Potential_Ecol_.

Following Cinner et al. [30], we used two techniques to examine social-ecological vulnerability. First, we developed a quantitative vulnerability score using an equation to combine the three contributing indices (each normalized to 0–1 scale) (Social-ecological vulnerability = [ecological exposure+social sensitivity] – social adaptive capacity). Secondly, to visualize differences in key components of vulnerability, we plotted the three dimensions on a bubble plot, where social sensitivity was plotted against social adaptive capacity and ecological vulnerability (i.e. social exposure) was indicated as the size of the points (larger point = higher exposure).

### Ethics Statement

JEC obtained ethics approval from James Cook University’s human research ethics committee (ID#H4331). We obtained verbal consent from participants before conducting surveys. During verbal consent, participants were informed about the survey, its purpose, and how the data would be utilized. Written consent from participants was not obtained because of low literacy rates in many of our field sites, which meant that participants may not have fully understood what they signed. Verbal consent was authorized by the James Cook University human ethics panel. Permission for social and ecological research in Kenya was provided by the National Secretary for Science and Technology (research clearance permit # NCST/RRI/12/1/BS/209).

## Results

### Ecological Aspects of Vulnerability

The ecological indicators were highly variable across the 15 study sites (Table S6 in File S1). Sites included degraded reefs with low coral abundance (<1% absolute live coral cover, Takaungu), limited coral diversity (13 genera, Kuruwitu), low reef fish biomass (<100 kg/ha, Kanamai, Takaungu, RasIwatine), limited herbivore grazing diversity (<0.01 Simpson index of Acanthurids, Siganids, and Scarids, Kanamai, RasIwatine), and herbivore grazing rates that were substantially less than estimated rates of algal production (>100 kg/day deficit, Mayungu, Takaungu). More intact reefs had higher coral cover (>50%, Mradi), more diverse coral assemblages (25 genera, Changai, Kisite), and more productive fish communities (∼1600 kg/ha reef fish biomass, Kisite) with greater herbivore diversity (∼0.7 Simpson index, Mombasa) and higher herbivore grazing relative to algal production (>50 kg/day surplus, Changai, Kisite).

**Figure 3:**
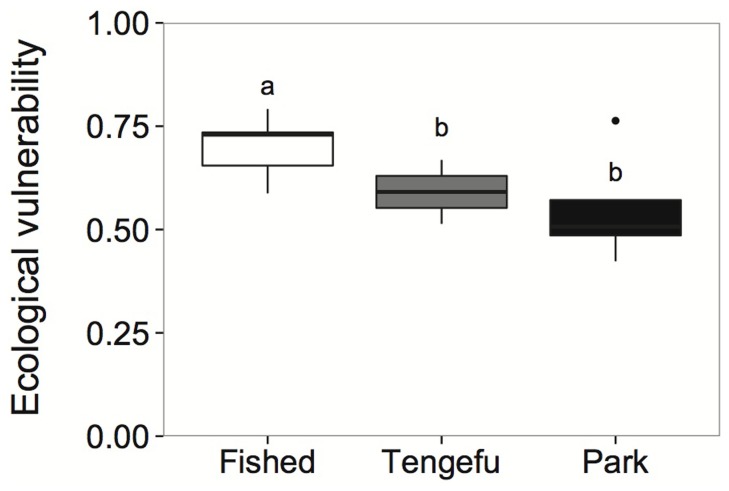
Ecological vulnerability on 17 Kenyan reefs across three types of fisheries management: open access fished reefs, community-managed ‘*tengefu*’, and National Marine Parks. One-way Analysis of Variance suggests fished reefs are marginally more vulnerable to climate change than *tengefu*s and no-take parks (one-way ANOVA, P = 0.08). Letters indicate where significant differences exist across management groups). The different colours of bars represent different management types corresponding to those in Figure 2.

The wide range of ecological condition across the 15 coral reef sites in Kenya led to considerable spread in the composite ecological vulnerability index (Table S6 in File S1, Figure 3). Ecological vulnerability ranged from 0.42 to 0.79 (mean 0.64±0.11 SD, vulnerability index scaled between 0 and 1). The three facets of ecological vulnerability (ecological exposure, ecological sensitivity and ecological recovery potential; Table S7 in File S1) were not strongly correlated, suggesting these different components of ecological resilience are not related (Pearson correlation coefficients: ecological exposure to ecological sensitivity, r = **−**0.46, ecological exposure to ecological recovery potential, r = **−**0.15, ecological sensitivity to ecological recovery potential, r = 0.11). Overall, fished sites had marginally higher ecological vulnerability than sites within tengefus and no-take marine reserves (one-way Analysis of Variance, F = 3.2, df = 2,12, *P* = 0.08; Table S7 in File S1; Figure 3).

**Figure 4:**
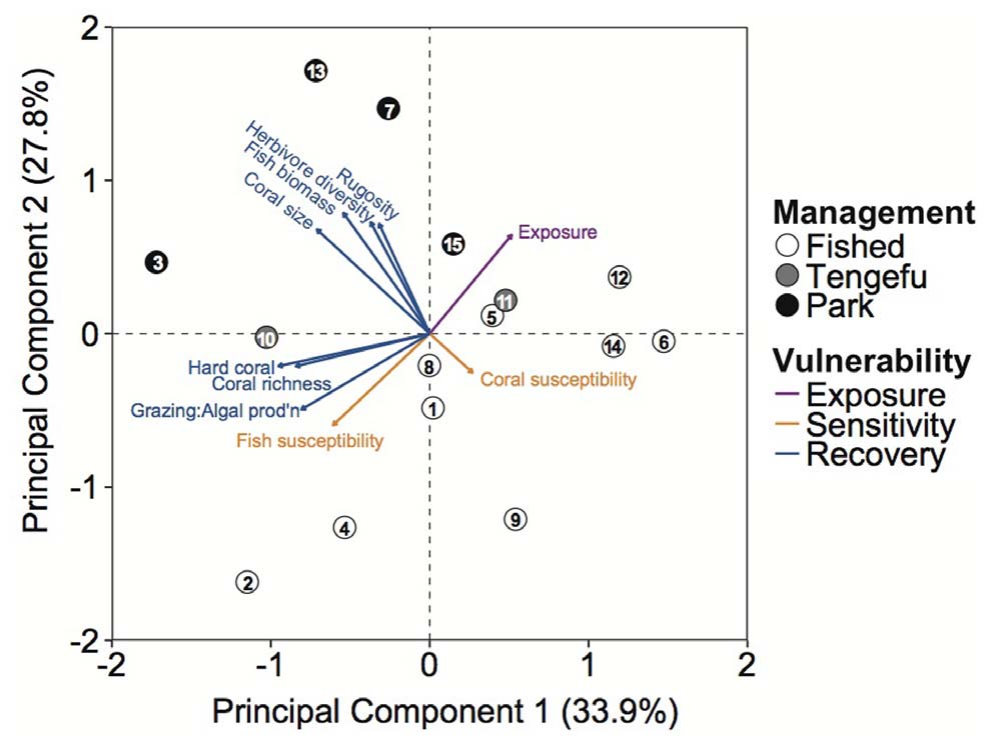
Principal components analysis of ecological vulnerability. Eigenvectors describe normalized indicators of exposure, sensitivity and recovery potential. Points indicate reefs within different management groups (white – fished; grey – community co-managed areas; black – no-take marine reserves). Numbers indicate study sites (see Table 1).

The two principal components axes explained 61.7% of the variation among indicators across the sites (Figure 4). Management did not distinguish exposure because some fished reefs, community-managed tengefus, and government no-take marine reserves were associated with high levels of exposure (upper-right quadrant of Figure 4). Fished reefs were associated with higher climate sensitivities of coral and fish assemblages (bottom quadrants). Recovery potential indicators separated into two groups. Herbivore diversity, rugosity, fish biomass, and coral size were associated with the no-take marine reserves (upper-left quadrant of Figure 4), while coral richness, hard coral cover, and higher rates of herbivore grazing relative to algal production were associated with one tengefu, Mradi, and some fished reefs (lower-left quadrant).

**Figure 5:**
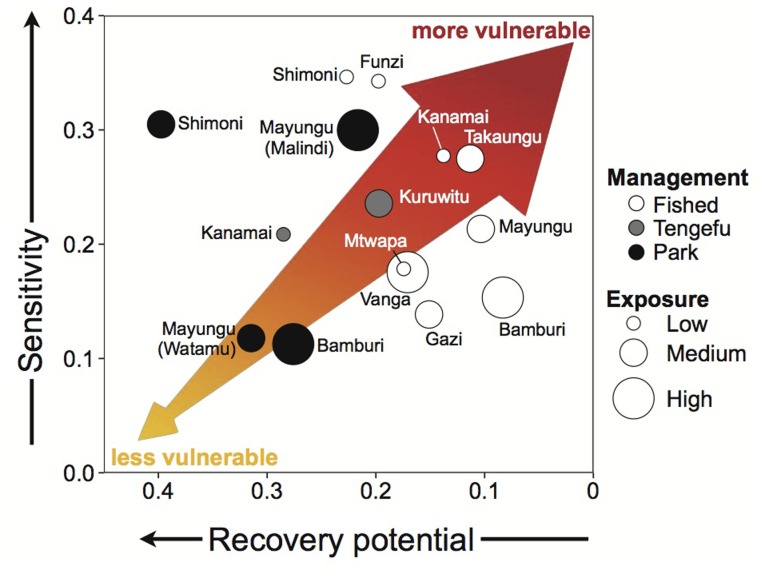
Ecological vulnerability of Kenyan coastal communities to the impacts of coral bleaching on reef fisheries. Ecological sensitivity is plotted against ecological recovery potential (note: axis is reversed) and ecological exposure is indicated by bubble size. The arrow highlights less vulnerable to more vulnerable communities.

There was a wide spread of the three facets of ecological vulnerability across different types of fisheries management. Variable exposure, high sensitivity, and low recovery potential to coral bleaching events resulted in higher ecological vulnerability scores for some fished sites, one tengefu (Kuruwitu) and some no-take marine reserves (upper right of Figure 5). Other no-take reserves and one tengefu (Mradi) were associated with lower ecological vulnerability due to low ecological sensitivity and high ecological recovery potential, despite medium to high exposure (Table S6 and S7 in File S1; Figure 5).

### Social Aspects of Vulnerability

#### Social sensitivity

We focused our sampling on direct resource users, meaning that the resource dependence aspects of sensitivity had relatively little variation between communities (ranging from 0.23–.35; Table S1 in File S1). Our analysis of the gear use side of sensitivity produced some counter-intuitive results and highlighted some key research gaps (Table S2 and S3 in File S1). In particular, we found that the sensitivity of certain gears types to the impacts of coral bleaching events varied considerably (Fig. S5 in File S1). In particular, the species targeted by traps and beach seine nets in the Kenyan fishery were expected to decline as a result of bleaching-induced mortality. However, available information to date suggested that the species targeted by other gears may actually demonstrate short-term increases in abundance as a result of bleaching mortality (Fig. S5 in File S1). However, we only had species-specific responses for 48–69% of the catch abundance (Fig. S3–S4, Table S4 in File S1) and many of the species-specific responses were supported by only one study (Fig. S6 in File S1).

#### Social adaptive capacity

The ten communities displayed considerable variation in many of the indicators of adaptive capacity that we measured (Table S8 in File S1), particularly access to credit, debt, human agency, capacity to change, social capital, community infrastructure, and material style of life. For example, the proportion of respondents not in debt (recorded as more than one week’s typical earnings) ranged from 55–90%. Alternatively, several of the indicators displayed little variation between the highest and lowest values, particularly, occupational mobility, gear diversity, and trust.

**Figure 6:**
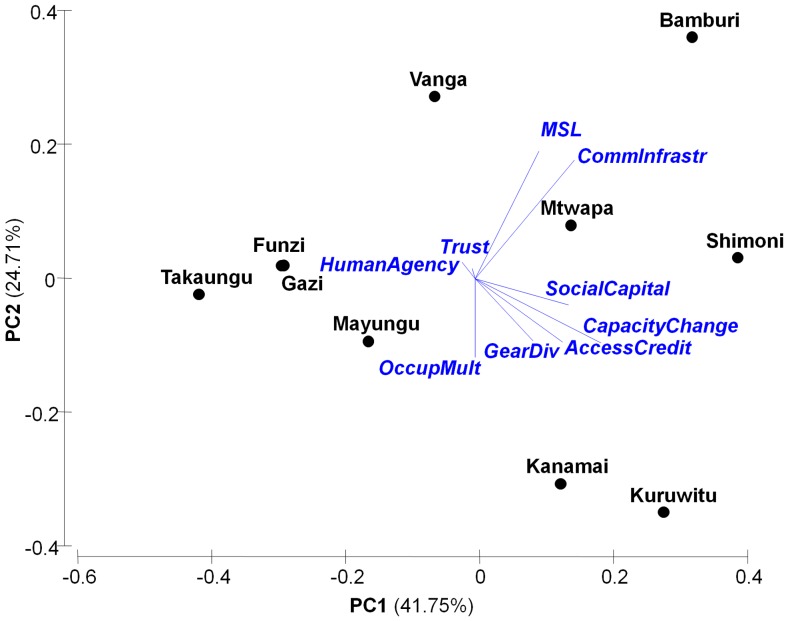
PCA of the 9 social adaptive capacity indicators analysed at an aggregate community level. The blue vectors represent the 9 social adaptive capacity indicators: Material style of life (MSL), Community Infrastructure (CommInfrastr), Trust, Social capital, Human Agency, Capacity to change (CapacityChange), Gear diversity (GearDiv), Access to Credit (AccessCredit) and Occupational Multiplicity (OccupMult) (No Debt and Occupational mobility not included). The black dots represent the 10 communities.

We ran a PCA based on the co-variance matrix (because the units were all on the same scale) (Figure 6). Visual inspection of scree plots revealed that the first three Principal Components (PCs), which explained 82% of the variance (Table S9 in File S1), could be used. Social capital, capacity to change, access to credit, community infrastructure, gear diversity, and Material Style of Life (MSL) all had substantial factor loadings on PC 1 (Table S10 in File S1). MSL, occupational multiplicity, and community infrastructure dominated PC2, but gear diversity and access to credit also had substantial loadings on that PC. Interestingly, MSL and community infrastructure loaded negatively on PC2, while gear diversity and occupational multiplicity loaded positively. Human agency loaded highly on the PC3 (Table S10 in File S1). Trust did not load highly on any of the components, primarily because there was little variation in trust between communities. Although there was substantial variation in trust at the individual level, community-level means and standard errors were relatively similar (Table S8 in File S1).

### Social-Ecological Vulnerability

**Figure 7:**
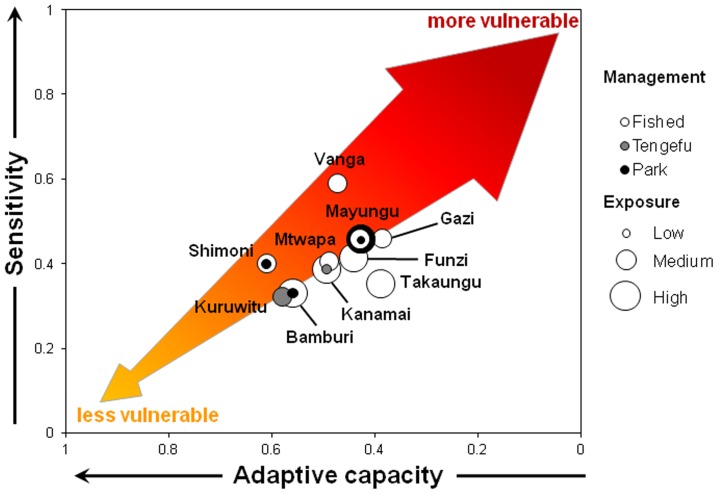
Social-ecological vulnerability of Kenyan coastal communities to the impacts of coral bleaching on reef fisheries. Social sensitivity is plotted against social adaptive capacity (note: axis is reversed) and ecological vulnerability is indicated by bubble size. The arrow highlights less vulnerable to more vulnerable communities. Note that some sites (such as Shimoni) may have more than 1 ytpe of management present, indicated by overlapping dots.

Our measure of social-ecological vulnerability comprised three components: 1) social exposure (which is ecological vulnerability; Fig. 1); 2) social sensitivity; and 3) social adaptive capacity. We used a bubble plot to visualize social-ecological vulnerability at our study sites (Figure 7). This visualization helped show how the vulnerability of our communities compared to each other and helped demonstrate which component(s) of vulnerability contributed the most to their vulnerability, so that specific actions could be taken for each of them. For example, Takaungu had a high vulnerability mainly due to its high exposure and low adaptive capacity, even though its sensitivity was low. Actions to improve the vulnerability of this community might focus on increasing adaptive capacity (it is harder to have actions that can reduce exposure). Vanga had a high vulnerability primarily because of its high sensitivity. Actions to improve the vulnerability of this community might focus on decreasing sensitivity.

## Discussion

Our modification to the IPCC vulnerability assessment framework provides a conceptual model for considering both socioeconomic and ecological dimensions in an integrated assessment of system vulnerability. Integration between socioeconomic and ecological systems highlights and exposes the codependency between the systems components; where vulnerability is visibly and quantitatively influenced by each component. Facing the growing threat of climate change and because of the inter-dependencies between people and ecosystems, understanding the linkages is increasingly important for effective management. Nevertheless, there are few examples quantifying this integrated understanding of vulnerability in the literature.

Integrated vulnerability analyses can be used to identify status, trends, and possible opportunities for adaptation in the face of climate change. In particular, our study exposes the role of local level management in influencing the sensitivity and recovery potential of corals and associated fish assemblages, which ultimately reduces exposure in the social domain. This is in contrast to ecological exposure, which can only be reduced by international action to reduce carbon emissions. Likewise, social adaptive capacity and social sensitivity are also amenable to policy actions at local and national scales [30].

This case study uses a diagnostic approach and supports the argument that one-size-fits-all or panaceas to adaptation planning are unlikely to succeed [44], [45]. Our methods and results highlight how specific aspects of adaptive capacity and sensitivity can determine the strengths and weaknesses that contribute to high or low vulnerability. This should be useful for adaptation planning that takes advantage of existing capacities and can strengthen the identified weaknesses. By examining the types of vulnerability (exposure, sensitivity and adaptive capacity) that different communities have (e.g. Figure 7), the most appropriate policy priorities become apparent (Table 3). For example, social systems might be buffered from ecological degradation through local management strategies that increase ecological recovery potential (e.g. through marine protected areas or gear-based management). Likewise, social sensitivity could be decreased by promoting the use of gears less likely to be negatively impacted by coral bleaching (e.g. handlines), or by creating supplemental livelihood activities. Adaptive capacity is perhaps the component of vulnerability most amenable to influence, and may be a useful focus for adaptation planning. Some aspects of adaptive capacity, such as infrastructure development, can be directly and predictably enhanced by physical development projects, while other livelihood or cognitive dimensions are not so amenable to enhancement by central government. Alternatively, some aspects of building adaptive capacity, such as skills development, support for micro-credit schemes, and poverty alleviation may be best delivered by NGOs and development organizations working in conjunction with local and national governments.

**Table 3:** Possible policy responses to influence different types of social-ecological vulnerability.

Vulnerability component	Potential to influence	Possible policy actions for enhancement
**Social Exposure (i.e. Ecological Vulnerability)**	Medium	Develop local level management to increase ecological recovery potential and ecological sensitivity (e.g. marine protected areas, gear based management).
**Social Sensitivity**		
Gear sensitivity	High	Promote the use of gears less likely to be negatively impacted by coral bleaching (e.g. hand lines)
Occupational sensitivity	Medium	Develop supplemental livelihood activities
**Social adaptive capacity**		
Capacity to Change livelihood	Low	Skills and capacity building
Access Credit	High	Microcredit schemes, support for community savings
Community Infrastructure	High	Infrastructure development projects in rural areas
Gear Diversity	Low	Training, gear provision
Trust	Low	Eradication of corruption
Occupational Multiplicity	Low	Support for economic growth
Wealth (MSL)	Low	Poverty alleviation plans and pro-poor growth policies
Recognition of Human Agency	Medium	Education and participation in research
Social Capital	Medium	Support for community initiatives/organizations

An important finding of our research highlights that there may be tradeoffs inherent in specific aspects of adaptive capacity, particularly those associated with people’s flexibility and assets. This finding is supported by studies of livelihood diversification, which have found occupational specialization with increasing socioeconomic development [35], [46]. Specialization within industries such as the fishing industry occurs as the result of capital being secured in vessels and equipment [47], [48]. This increases the efficiency of the operation, decreases the price of the product, and maintains social status [46]. Yet, resource-users that target only a few species, or are reliant on a single resource are severely restrained in their ability to be flexible and adapt to changes in the resource relationship. Specialist behaviour is typical of regions in which resources are abundant and predictable and the system is regarded as ‘stable’. However, the ‘stable’ system is not necessarily resilient in the face of change. Thus, in areas where resources are less predictable, a ‘generalist’ or risk-spreading strategy may be more resilient. Generalists or resource-users that target more than one species can exhibit a more flexible nature since they can interchange between resource types as the need arise.

A surprising result from this study is that, based on available information to date, it appears as though temperature-induced coral mortality has the potential to result in modest short-term increases in catches for some gear users. For example, the algae that often grow over dead corals could promote the abundance of certain types of low-trophic herbivorous fishes that are targeted by certain gears. Thus, sensitivity is not always negative; climate change could impact some fish species, some gear, and some people positively. However, a degraded and algal covered reef is unlikely to sustain fish populations after the structural complexity of the reef has declined. Likewise, targeting the species that eat algae may hinder prospects of reef recovery after a bleaching event. Thus, we do not view these potential selective short-term increases in catch as a sustainable fisheries benefit from climate change. Additionally, our initial investigation of the impacts of temperature-induced mortality on reef fishers examined likely changes to *in situ* abundance of commonly targeted reef fishes, but does not consider status or trends of key fisheries parameters such as catch per unit effort, biomass, trophic structure, or catchability that are often used to estimate yields. Future studies may incorporate these types of fisheries parameters in estimates of gear sensitivity. Lastly, our results here should not be generalized to how other reef fisheries may respond to further bleaching events. Our analysis could produce extremely different results somewhere like Papua New Guinea, where many of the species captured by artisanal fishers are more reef associated and the starting condition of the fishery is much better [41], [43]. A limitation of the approach we employed is that we were unable to examine changes in catch sensitivity over time. A key concept in fisheries is that catch compositions can change over time.

Our study is the most comprehensive of its kind, particularly for reef fisheries. However, there are several caveats about our approach and methodology that are important to acknowledge. We are aware that our index of vulnerability is limited to the effect of a single climate change impact (coral bleaching), through a single impact pathway (impact on fisheries). In reality, climate change is a multifaceted threat that will comprise multiple interacting impacts that will also be mediated or extenuated by other social and economic trends. The impacts of climate change on fisheries through coral bleaching are hard to discern and may be overwhelmed by: i) existing trends such as overexploitation; ii) climate impacts affecting other aspects of the ecosystem (e.g. seagrasses); iii) or socioeconomic characteristics, such as demographics, migration and the provision of food and employment from agriculture. In addition, the novel indexes we use here incorporate multiple sources of uncertainty about the nature of exposure, sensitivity and adaptive capacity and this high level of uncertainty needs to be recognized by adaptation prioritization and planning efforts.

Our methodology also assumes future sensitivity and adaptive capacity based on a snapshot of current conditions. Clearly, climate impacts or other external forces such as development projects (e.g. the proposed port development project in Lamu in northern Kenya) could result in substantial economic and social restructuring of surrounding coastal communities in ways that would profoundly alter social sensitivity and social adaptive capacity. Likewise, this study focuses on impacts on currently targeted species, which could be altered by climate change. For example, climate anomalies in Peru that severely impacted the dominant anchovy fishery also created opportunities for exploitation of different species in different areas, which were taken up fishers who had spatial and technological flexibility to exploit them [49]. Additionally, our ecological research is focused on coral reef fish species, although certain non-coral associated (e.g. *Leptoscarus vaigiensis* and *Siganus sutor*), pelagic and semi-pelagic (e.g., *Sphyraena barracuda*), and non-fish resources (e.g. lobsters and octopus) are also significant fishery resources supporting livelihoods and food security. Despite these caveats, we present a first step to understanding vulnerability, and highlight the importance of maximizing use of all available data when assessing the vulnerability of a place.

This study advances the application of climate change impact and adaptation theory to empirical data, and identifies several key gaps requiring further research. For example, our socioeconomic study focused on direct resource users with only limited information about the broader socioeconomic context, which can be a significant driver of social adaptive capacity. An understanding of the broader socioeconomic context within which resource users are embedded may further progress our understanding of how resource dependent people can be assisted so as to minimise their vulnerability to future climate changes. Similarly, the relative importance of different components of adaptive capacity for adapting to different types and magnitudes of impacts over time is not well understood. For example how can we understand the tradeoff between infrastructure and wealth resources with development and the loss of occupational flexibility? We also recognize that future research will need to consider the susceptibility of fish to climate impacts other than coral bleaching (e.g. ocean acidification), and there is a need to ascertain the species-specific responses to bleaching of five key fishes that makes up a large proportion of the catch (Methods S1 in File S1).

## Conclusions

The modified IPCC vulnerability assessment framework provides a useful model for assessing adaptive capacity and sensitivity of social systems that are exposed to changes in the condition of the ecological system upon which they depend. In applying this modified model to resource-dependent communities in Kenya, we are able to derive useful insights into the relative magnitude and key sources of vulnerability to potential climate changes and to consider possible strategies that can minimise vulnerability. The framework allows us to simplify assessments and consider heterogeneity within: 1) places that experience climate change-related events such as coral bleaching; 2) the ways that coral reef ecosystems are affected by and can recover from these impacts; 3) the ways that societies and individuals are impacted by these changes; and 4) the capacity of people to cope with and adapt to these changes. Overall, indicators of ecological exposure, ecological sensitivity, and ecological recovery potential are different facets of ecological vulnerability, which provides justification to our effort to identify indicators describing these different aspects of the vulnerability. Although focusing on small-scale fisheries that operate in coral reef systems, the vulnerability assessment, framework, and survey we develop are adaptable to other kinds of fishery or natural resource dependent systems. Likewise, the framework could be adapted to explore vulnerability to other kinds of environmental, economic, or social stresses and could be complemented by qualitative social science research methodologies [50], [51], [52].

## Supporting Information

File S1 **Contains: Methods S1. Figure S1.** Ecological indicators compared across sites in the western Indian Ocean sites (n = 482), Kenya (n = 214), and the 15 Kenyan sites included in this study (Labelled Kenya BMU in this figure). Box plots show 25% and 75% quartiles (box) with median (line) and outliers. **Figure S2.** Comparison between indicator values normalized to Kenya 2% and 98% percentiles, vs. Western Indian Ocean regional site 2% and 98% percentiles. The red line indicates the 1∶1 line. **Figure S3.** Relative contribution in fish abundance from catch data of species, genus, family level data and species with no data. F**igure S4.** Relative abundance of species targeted by gear type. Species are coloured as to whether we have species level data (black), genus level averages (dark grey), family level averages (light grey), or no data (white) on their response to coral mortality. **Figure S5.** Average fish response to coral decline of each gear using only species data, or species and genus data, or species, genus and family data, ±SE. **Figure S6.** Relative abundance *response to decline of fish species targeted by gear type. This figure illustrates the influence of each species on the results and helps to identify critical research directions. The colour indicates the number of study in the global database of species response to coral loss that were used for each species: green for more than 1 study, red for only 1 study, and black where genus data were used. **Figure S7.** Intergovernmental Panel on Climate Change (IPCC) conceptual framework of vulnerability to climate change. **Table S1.** Occupational sensitivity scores by community. A score of 1 would mean all respondents depended on marine resources and had no livelihood alternatives, while a score of 0 would mean that none of the respondents had marine resource based livelihoods. **Table S2.** Average percent change in abundance of fish per percent decline in coral cover by gear type, using species and genus data (and also without Lethrinus nebulosus). **Table S3.** Gear sensitivity scores by community. **Table S4.** Missing information on five species creates a significant gap in our understanding on how species respond to coral mortality. Column 1 shows the relative abundance of the five critical species without species-specific data on responses to coral mortality by gear type. Column 2 shows existing species level data by gear type. Column 3 shows the proportion of catch data that we would have species-specific understandings of if just five species were studied. **Table S5.** Spearman correlations between the 11 adaptive capacity indicators (correlations conducted at the community scale). **significant at 0.01, *significant at 0.05. **Table S6.** Ecological vulnerability indicators of exposure, sensitivity and recovery potential for 15 ecological sites. Detailed description of the rational for indicators and how indicators were calculated can be found in Table 1 and the Methods. **Table S7.** Dimensions of ecological vulnerability for 17 coral reef sites in Kenya. Ecological vulnerability was calculated from normalized and weighted indicators as (Exposure+Sensitivity) – Recovery Potential. Sites are ranked from most vulnerable to least vulnerable. **Table S8**. The 11 adaptive capacity indicators aggregate values at community level shown as % or mean ± standard deviations. **Table S9.** Eigenvalues and percentage of variation explained by the different PCs**. Table S10.** Factor loadings of adaptive capacity indicators. Factor loadings above 0.4 (in bold) on any given Principal Component are generally considered to contribute substantially to that Component. **Table S11**. Absolute factor loadings, weights and normalised weights of each adaptive capacity indicator.
(DOCX)
