# Supplementary material for: Evaluating Social and Ecological Vulnerability of Coral Reef Fisheries to Climate Change
Source: PLoS One. 2013 Sep 11;8(9):e74321. doi: 10.1371/journal.pone.0074321 (PMC3770588; doi:10.1371/journal.pone.0074321)
Supplement: File S1 — Contains: Methods S1. Figure S1. Ecological indicators compared across sites in the western Indian Ocean sites (n = 482), Kenya (n = 214), and the 15 Kenyan sites included in this study (Labelled Kenya BMU in this figure). Box plots show 25% and 75% quartiles (box) with median (line) and outliers. Figure S2. Comparison between indicator values normalized to Kenya 2% and 98% percentiles, vs. Western Indian Ocean regional site 2% and 98% percentiles. The red line indicates the 1∶1 line. Figure S3. Relative contribution in fish abundance from catch data of species, genus, family level data and species with no data. Figure S4. Relative abundance of species targeted by gear type. Species are coloured as to whether we have species level data (black), genus level averages (dark grey), family level averages (light grey), or no data (white) on their response to coral mortality. Figure S5. Average fish response to coral decline of each gear using only species data, or species and genus data, or species, genus and family data, ±SE. Figure S6. Relative abundance *response to decline of fish species targeted by gear type. This figure illustrates the influence of each species on the results and helps to identify critical research directions. The colour indicates the number of study in the global database of species response to coral loss that were used for each species: green for more than 1 study, red for only 1 study, and black where genus data were used. Figure S7. Intergovernmental Panel on Climate Change (IPCC) conceptual framework of vulnerability to climate change. Table S1. Occupational sensitivity scores by community. A score of 1 would mean all respondents depended on marine resources and had no livelihood alternatives, while a score of 0 would mean that none of the respondents had marine resource based livelihoods. Table S2. Average percent change in abundance of fish per percent decline in coral cover by gear type, using species and genus data (and also without Lethri [file pone.0074321.s001.docx]

# Methods S1

# Ecological methods

We quantified coral reef habitat using 10 m line intercept transects (n = 4 - 9 transects per site). The lengths of major benthic components (hard coral, soft coral, turf algae, macroalgae, and crustose coralline algae) underlying each transect line were measured to the nearest centimeter. Percent cover was calculated as the sum of the lengths of each benthic group divided by the total transect length. Hard corals were identified to genus, and the genus *Porites* was subdivided into three distinct morphological groups: massive *Porites*, branching *Porites* and a subgenus *Synaraea* (*Porites rus*).

Hard coral communities were also evaluated using roving observer surveys to quantify coral genera richness and community structure over a larger reef area. On each survey, an observer haphazardly delineated ~20 2m^2^ quadrats and within each quadrat identified coral colonies to genus and scored each colony for observed bleaching intensity and mortality on a six-point scale (*c*_0_ = normal, *c*_1_ = pale live coral, *c*_2_ = 0-20%, *c*_3_ = 20-50%, *c*_4_ = 50-80%, *c*_5_ = 80-100% of the live coral surface area fully bleached, and *c*_6_ = recently dead). Estimates of bleaching occurrence and the relative abundance of hard coral genera were used to estimate the bleaching susceptibility of the coral community (see Ecological Indicators below).

Reef fish communities were surveyed using 2 – 4 replicate 5 x 100 m belt transects at each site. Individuals were identified to family and estimated into 10-cm size class bins. Wet weight biomass per family were estimated from length – weight correlations established from measurements of the common species in each family taken at local fish landing sites in Kenya [[1](#_ENREF_1)]. Total reef fish biomass was calculated as the sum of family wet weights on each transect. We also estimate species richness and abundances of the fish community from the number of observed species in four species families (Acanthuridae, Chaetodontidae, Labridae, and Scaridae). Species richness estimates were then standardized and expressed as the number of species per 500 m^2^. We have commonly used this method to survey reef fish species richness and is expected to be a useful proxy for the total number of reef fish species present [[2](#_ENREF_2)].

Within the Kenyan study sites, four indicators of recovery potential (coral:macroalgae cover, calcareous:non-calcareous cover, fish size CV and fish species richness) were highly collinear as identified from Pearson correlation coefficients with the other recovery indicators and variance inflation factors. These variables were removed from further analysis to prevent bias within the composite recovery potential metric. Importantly, the ecological processes represented by the four excluded indicators were represented by other variables that remained in the analysis.

**Exposure** to the impacts of coral bleaching was estimated using a previously published spatial model of susceptibility to bleaching-induced mortality [[3](#_ENREF_3),[4](#_ENREF_4)]. The model evaluated the relationships between the prevalence of coral bleaching [based on reported intensity of bleaching from observations available in ReefBase [[5](#_ENREF_5)] and 11 different environmental conditions. Each environmental variable was weighted by the strength of the environmental factor-bleaching relationships and all factors summed and normalized. This provided us with a site-specific exposure or index of bleaching stress.

**Coral bleaching susceptibility** was estimated from the coral community structure estimated on roving observer surveys, weighted by the regional taxa-specific bleaching sensitivity of each genus [[6](#_ENREF_6),[7](#_ENREF_7)]. The bleaching response of each genus in the western Indian Ocean was calculated from 141 surveys (n = 48,798 coral colonies) that occurred during bleaching events (i.e., where > 10% of the coral colonies at a site displayed bleaching) at 125 sites in 10 countries (Comoros, Kenya, Madagascar, Maldives, Mauritius, Mayotte, Mozambique, Reunion, Seychelles, South Africa and Tanzania) over 7 years (1998, 2004, 2005, 2007-2010). Bleaching susceptibility of coral communities was estimated at each site based on the relative abundance of coral taxa and their observed bleaching response:

Where *RA* is the relative abundance of each coral taxon, *i*, multiplied by its taxon-specific bleaching response, *BR_i_*, and then summed across all observed taxa at a site. **Reef fish susceptibility** at each site was similar to the coral susceptibility index, in that the relative abundance of each species, *j*, was multiplied by a taxon-specific climate vulnerability index (*V*_climate_) and then summed across all species observed at a site to provide a site-level estimate of the vulnerability of the reef fish assemblage to habitat loss associated with coral bleaching.

Climate vulnerability for reef fishes was assessed by Graham et al. [[8](#_ENREF_8)] from four variables that are known to relate to fish population declines following coral bleaching and mortality: diet specialization, habitat specialization, recruitment specialization to live coral and body size.

**Ecological recovery potential** was estimated by developing a weighted metric based on 9 ecological indicators. **Hard coral cover** was estimated as the average percent cover of live coral from replicate transects at each site. **Coral to macroalgae cover** was calculated as the ratio of hard coral cover to the combined cover of fleshy macroalgae and turf algae. Calcifying to non-calcifying cover was calculated as the ratio of the combined cover of hard corals, crustose coralline algae and calcareous algae (e.g., *Halimeda* spp.) to the combined cover of fleshy macroalgae and turf algae. **Coral size distribution** was estimated as the coefficient of variation (CV, mean size / standard deviation of size) of the average size of each coral genus at a site. Higher coral size CV values indicate more evenly sized coral assemblages with smaller recruits, juvenile corals and larger colonies of more mature adults. Lower values of coral size CV indicate assemblages that do not have an even distribution across size classes, which may indicate either recruitment limitation (i.e., few recruits and juvenile corals) or limited adult reproductive stock (i.e., few large reproducing adult colonies). We calculated **coral richness** as the number of genera observed in the community from roving observer surveys, a method that surveys more reef area and can provide a more accurate estimate of coral diversity than line intercept transects (T. McClanahan and E. Darling, unpublished data).

**Fish biomass** (kg ha^-1^) was calculated as total wet weight of all surveyed reef fishes from replicate 5 x 100m belt transects at each site (see ecological sampling methods). **Species richness** of fishes was also calculated from replicate belt transects as the total number of species per 500 m^2^ in four surveyed families (Acanthuridae, Chaetodontidae, Labridae, Scaridae). **Substrate complexity** (or rugosity**)** was calculated on each transect using the standard measure of the contour of the habitat over 10 m divided by the straight-line distance under the contour; replicate transect rugosity values were then averaged to estimate site-level rugosity. We estimated **fish size distribution** as the coefficient of variation of family level fish abundances measured to 10 cm bins. **Herbivore diversity** was estimated from energetic-based grazing rate of three herbivorous fish families (Acanthuridae – surgeonfishes; Scaridae – parrotfishes; Siganidae – rabbitfishes) and sea urchins. Herbivorous fishes and sea urchins have been reported to consume 22% and 2% of their body mass per day, respectively [[9](#_ENREF_9),[10](#_ENREF_10)]. We calculated the average algal consumption (kg day^-1^) for each of the four major herbivore groups (acanthurids, scarids, siganids and sea urchins) and calculated the Simpson diversity index as a functional estimate of herbivore grazing diversity. Finally, we quantified the amount of **herbivore grazing relative to algal production** as the difference between the total herbivore grazing rates on algae (fishes and sea urchins; kg ha^-1^ day^-1^) and the rate of algal production (kg ha^-1^ day^-1^) at each site. To estimate algal production, we used an estimate of gross algal production of 196 kg ha^-1^ day^-1^ at 100% algal cover [[9](#_ENREF_9),[10](#_ENREF_10)] multiplied by the observed average percent cover of algae (turf, macroalgae, calcareous and coralline algae) estimated at each site from coral habitat transects.

For each indicator of exposure, sensitivity and recovery potential, we calculated values for the 15 ecological study sites and used box plots to compare how these values were distributed among sites studied along the entire Kenyan coastline (n = 214), as well as sites from regional surveys throughout the western Indian Ocean (n = 482) (Figure S1). This enabled the range of values from the current Kenya study to be put in a broader Kenyan and regional context to assess how representative of extreme values the data are (Figure S1).


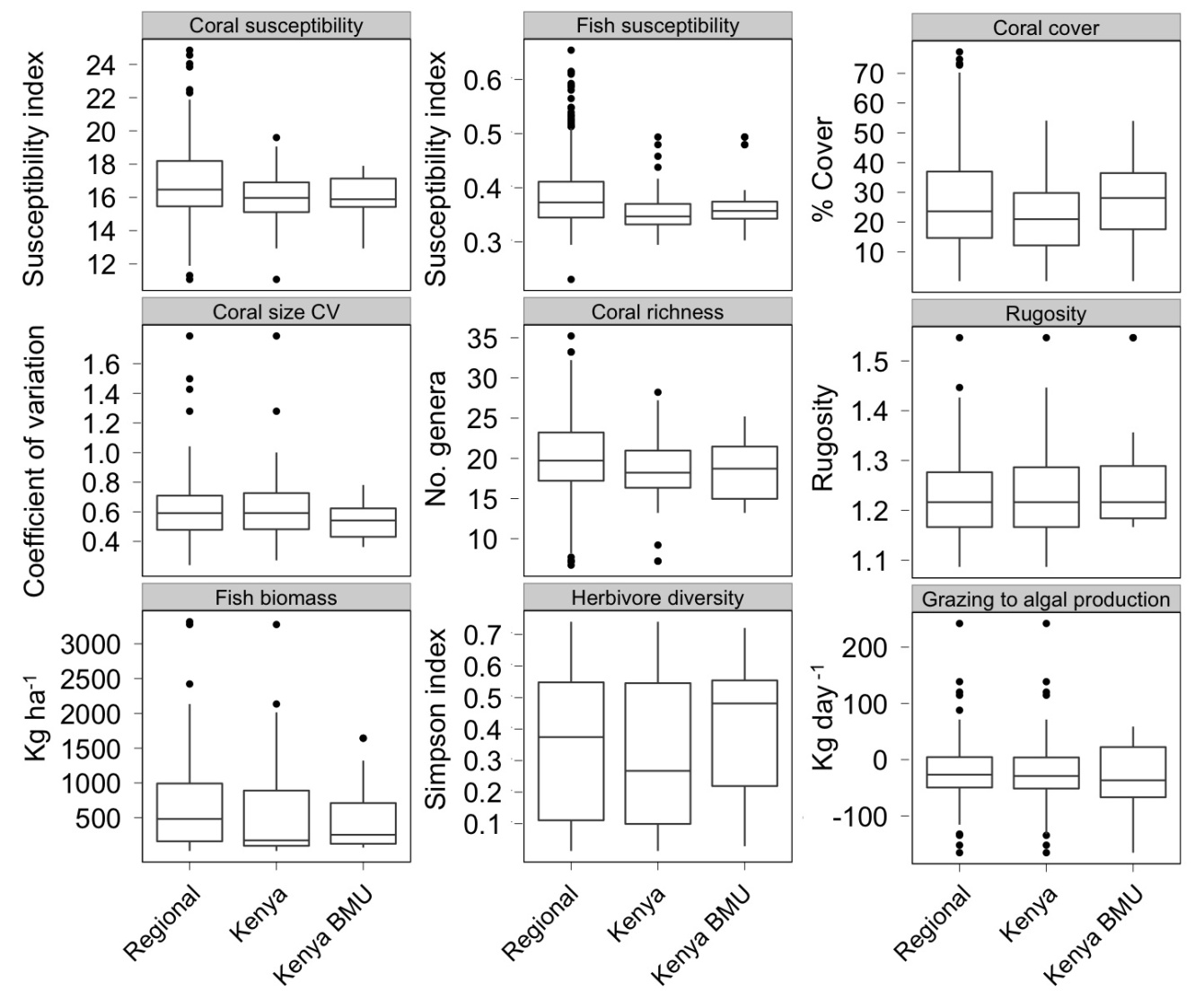


Figure S1. Ecological indicators compared across sites in the western Indian Ocean sites (n = 482), Kenya (n = 214), and the 15 Kenyan sites included in this study (Labelled Kenya BMU in this figure). Box plots show 25% and 75% quartiles (box) with median (line) and outliers.

**Normalizing the data**

For each indicator, we normalized values in two ways, both of which aimed at bounding the ecological variables within a broader geographic variation. First, to 2% and 98% percentiles from 214 Kenyan sites and second, to 2% and 98% percentiles from 482 western Indian Ocean sites. Percentiles were used as ‘minimum’ and ‘maximum’ estimates to ‘bound’ the site-level variables between 0 and 1 and are a better estimate of ‘true’ ecological minimum and maximum values and not potentially biased by influential outliers. Across all indicators, the normalized values using the Kenyan bounds were positively correlated to the indicator values normalized to the WIO regional bounds (linear regression, *R^2^* = 0.85, *P* < 0.0001). In general, the regional WIO range of values was greater than the Kenyan range of values, although generally these two bounding estimates are fairly similar (Figure S1and Figure S2). We used the regional bounds for normalizing the indicators for further analyses to ensure the current study is framed in a larger geographical context.


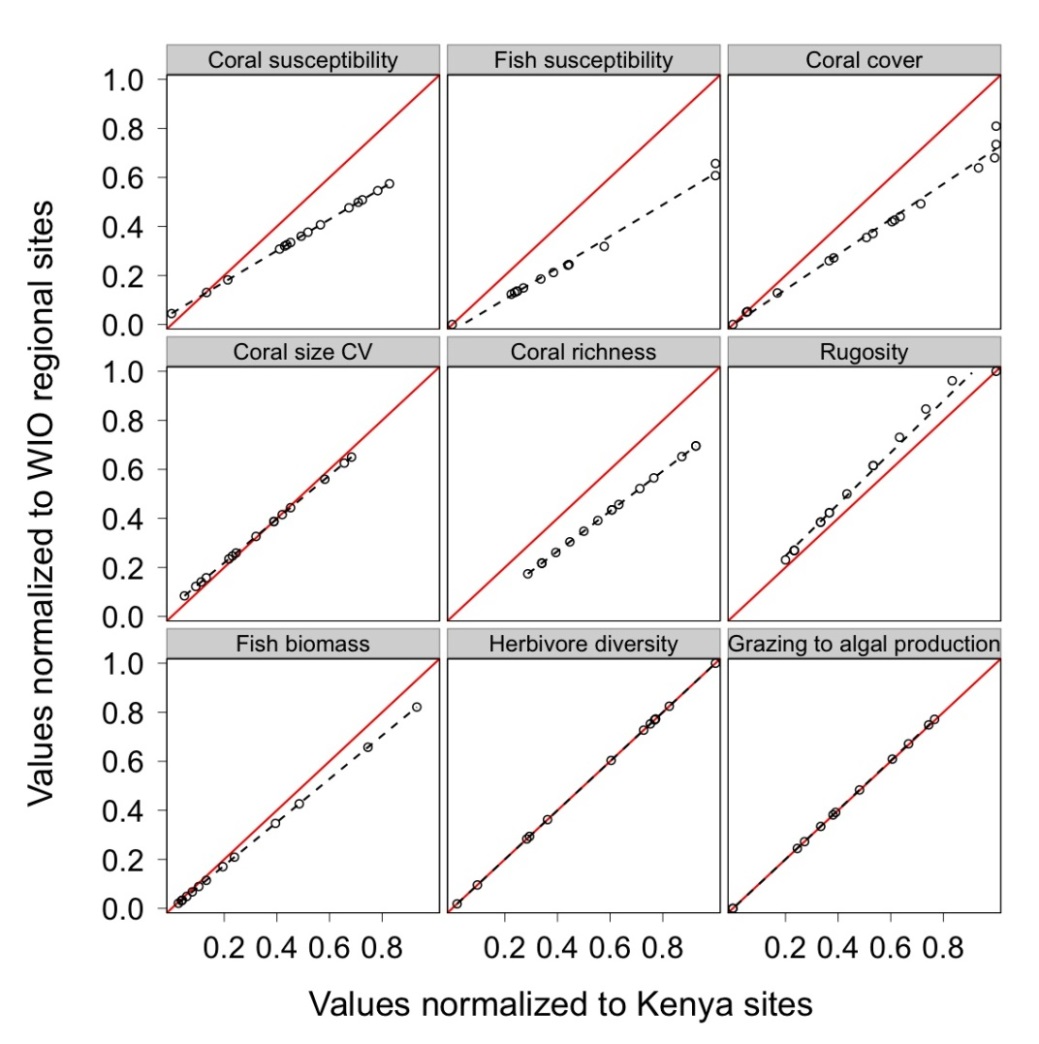


Figure S2. Comparison between indicator values normalized to Kenya 2% and 98% percentiles, vs. Western Indian Ocean regional site 2% and 98% percentiles. The red line indicates the 1:1 line.

**Social vulnerability methods**

***Sensitivity***

To develop the dependence component of the sensitivity metric, we asked respondents to list all livelihood activities that bring in food or income to the household and rank them in order of importance. Occupations were grouped into the following categories: fishing, selling marine products, gleaning, mariculture, tourism, farming, cash crops, salaried employment, the informal sector, other, and ‘none’ [[see 11 for details](#_ENREF_11)]. To better understand sensitivity to the impacts of temperature events on fisheries, we considered fishing, fish trading, gleaning, and mariculture together as the ‘fisheries’ sector and all other categories as the ‘non-fisheries’ sector. Our metric of sensitivity incorporates the proportion of households engaged in fisheries, whether these households also engage in non-fisheries occupations (what we call ‘linkages’ between sectors), and the directionality of these linkages (i.e. whether respondents ranked fisheries as more important than, say, agriculture) (Equation 1). We used the bracketed part of equation 1 to develop an occupational sensitivity score for each community (Table S1).

Equation 1

Where S=sensitivity, F=number of households relying on fishery-related occupations, NF=number of households relying on non-fishery-related occupation, N = Number of households, r_fn_=The number of times fisheries related occupations were ranked higher than non-fishing occupations (normalized by the number of households), r_nf_= The number of times non-fisheries related occupations were ranked higher than fishery occupations (normalized by the number of households), and G is the susceptibility of each specific gear type used (described below), and n is the number of gears. In the first bracket of Equation 1, the first term captures the ratio of fishery to non-fishery related occupations. The second term captures the extent to which households dependent on fisheries also engage in non-fishery livelihood activities. This term decreases the level of sensitivity when many households are engaged in both occupational categories. The third term captures the directionally of linkages between fisheries and non-fisheries such that communities were more sensitive when households engaged in fisheries and non-fisheries occupations consistently ranked the fisheries sector as more important than other livelihood activities. Using the bracketed part of the sensitivity equation provides a measure of occupational sensitivity (Table S1). The fourth term (i.e. top, outside the bracket) captures the selectivity of fishing gears and the differential impacts this may have on sensitivity to climate change.

Table S1. Occupational sensitivity scores by community. A score of 1 would mean all respondents depended on marine resources and had no livelihood alternatives, while a score of 0 would mean that none of the respondents had marine resource based livelihoods.

| Community | Occupational sensitivity |
| --- | --- |
| Bamburi | 0.32 |
| Funzi | 0.28 |
| Gazi | 0.27 |
| Kanamai | 0.34 |
| Kuruwitu | 0.23 |
| Mayungu | 0.3 |
| Mtwapa | 0.34 |
| Shimoni | 0.26 |
| Takaungu | 0.27 |
| Vanga | 0.35 |

The second part of our sensitivity index used data from a global database on species-specific responses of fishes to coral decline [[12](#_ENREF_12)], and catch records from Kenya [[13](#_ENREF_13),[14](#_ENREF_14)] to determine the use of which specific fishing gears might make people more or less sensitive to coral bleaching. Catch abundance data were collected at landing sites between October 2004 and May 2008 with a lesser amount collected in 1998. Where possible the entire catch was sampled, but when this was not possible a sub-sample was taken, ensuring that each gear used at each site was sampled and each species landed was recorded. Each of the 4205 fishes was identified to species level [[15](#_ENREF_15)]. For each catch, we recorded the gear used by the fisherman. This allowed us to ascertain the species selectivity for each gear type. We then integrated this gear selectivity data from Kenya with a global database on species-specific responses of fishes to coral bleaching, which provides a rate of decline per standardized percent loss of coral cover [[12](#_ENREF_12)]. We had data on species-specific responses to bleaching for 90 of the 265 species in our catch records (Figure S3 and Figure S4). We then entered the standardized response for each species into our catch records and pooled this by gear type. This allowed us to determine how gears selectively target species that have been shown to decline from coral bleaching, and provided us with a single value of mean expected decline for each gear. As described below, we used genus level averages when species-specific information was not available, and also dropped one species that had a disproportionate influence on the results. We then used an inverse of the response to coral decline by gear type (Table S2) to create a sensitivity measure for each gear. This resulted in negative sensitivity scores if the assemblage of gears used were likely to have positive effects on catch and positive scores if the yields were likely to be negatively affected. To create a gear vulnerability score for each community, we multiplied the gear usage (based on our survey data) by the gear vulnerability (Table S3).

Figure S3. Relative contribution in fish abundance from catch data of species, genus, family level data and species with no data.

|  |  |
| --- | --- |

Figure S4. Relative abundance of species targeted by gear type. Species are coloured as to whether we have species level data (black), genus level averages (dark grey), family level averages (light grey), or no data (white) on their response to coral mortality

Table S2. Average percent change in abundance of fish per percent decline in coral cover by gear type, using species and genus data (and also without Lethrinus nebulosus).

| Gear | Average Response to coral decline |
| --- | --- |
| Beach seine | -0.29 (±0.08) |
| Line | 0.60 (±0.09) |
| Net | 0.27 (±0.10) |
| Spear | 0.17 (±0.09) |
| Trap | -0.08 (±0.06) |

Table S3. Gear sensitivity scores by community

| Community | Beach seine (%) | Line (%) | Net (%) | Spear (%) | Trap (%) | Other (%) | Community aggregate of gear sensitivity to coral decline (inverse of response to decline) |
| --- | --- | --- | --- | --- | --- | --- | --- |
| Bamburi | 0.0 | 36.4 | 54.5 | 0.0 | 13.6 | 18.2 | -0.30 (±0.18) |
| Funzi | 11.1 | 27.8 | 11.1 | 0.0 | 11.1 | 72.2 | -0.11 (±0.24) |
| Gazi | 35.5 | 0.0 | 45.2 | 3.2 | 6.4 | 16.1 | -0.02 (±0.23) |
| Kanamai | 0.0 | 5.9 | 47.1 | 58.8 | 5.9 | 5.9 | -0.22 (±0.08) |
| Kuruwitu | 0.0 | 7.4 | 66.7 | 40.7 | 0.0 | 33.3 | -0.23 (±0.06) |
| Mayungu | 33.3 | 20.8 | 20.8 | 0.0 | 12.5 | 25.0 | -0.05 (±0.26) |
| Mtwapa | 14.8 | 11.1 | 63.0 | 25.9 | 3.7 | 14.8 | -0.19 (±0.18) |
| Shimoni | 0.0 | 20.8 | 20.8 | 8.3 | 50.0 | 54.2 | -0.12 (±0.10) |
| Takaungu | 8.3 | 8.3 | 62.5 | 25.0 | 0.0 | 16.7 | -0.20 (±0.16) |
| Vanga | 66.7 | 3.7 | 11.1 | 0.0 | 11.1 | 22.2 | 0.14 (±0.24) |

We were interested in whether we could use genus or family level averages of these species-specific responses as a surrogate, to fill in the missing data. Genus-level averages added another 15-20% to our catch records, and we had family-level information on vulnerability for about 90% of our catch records. We explored how using genus or family averages might change our responses, and also whether our data were heavily influenced by one particular species (Figure S5). The estimates did not change significantly when genus level averages were used to fill in missing data; however family level averages did change the results considerably (Figure S5). Consequently, we used genus level surrogates where data were available. Additionally, one species in particular stood out as having a very strong influence on our data and was consequently removed from subsequent analyses. *Lethrinus nebulosus* was heavily caught by many of the gears (Figure S4), but the changes in abundance relative to coral loss were extremely high (a 12% increase per percentage of coral loss, Figure S6), which came from only one study in the global database of species response to coral loss [[12](#_ENREF_12)]. Because the abundance changes were so high and this result was from only one study in Seychelles, we decided to drop this species from further analyses (Figure S5).

Figure S5. Average fish response to coral decline of each gear using only species data, or species and genus data, or species, genus and family data, ±SE

Our initial investigation indicated how different types of fishers might be affected by or benefit from expected changes to coral reefs. Surprisingly, based on species-specific plus genus level average responses to declining coral cover (and not including *Lethrinus nebulosus*), we found that the only gears that showed a likely decrease in catch were traps and beach seines (0.08 and 0.29% respectively; Figure S5, Table S2). Surprisingly, the other three gears actually showed a potential for a small increase in catch with coral mortality. This is largely because, in Kenya, fishers use a mosaic of habitats and many of the most commonly caught species are associated with sea grass and algae; habitats that would be unaffected by, or possibly benefit from coral mortality. Interestingly, line fishing showed a potential for a substantial (0.6%) increase in abundance of target species per percent loss in coral cover. One caveat to our analysis is that the Kenyan reefs are highly degraded and the lagoon fishery is heavily overfished. Consequently, the catch consists of many short-lived species that depend on sea grass and algae. Critically, our results here should not be generalized to how other reef fisheries may respond to further bleaching events. Our analysis could produce extremely different results somewhere like Papua New Guinea, where many of the species captured by artisanal fishers are more reef associated and the starting condition of the fisheries are often much better [[13](#_ENREF_13),[16](#_ENREF_16)].

Figure S6. Relative abundance * response to decline of fish species targeted by gear type. This figure illustrates the influence of each species on the results and helps to identify critical research directions. The colour indicates the number of study in the global database of species response to coral loss that were used for each species: green for more than 1 study, red for only 1 study, and black where genus data were used.

A limitation of this approach is that it did not examine changes in catch sensitivity over time. A key concept in fisheries is that catches change over time. Often, the species most vulnerable to overfishing are caught first, and as a system becomes more overfished, less vulnerable species are targeted (because the more vulnerable ones have been removed). Our study used a static estimate for species composition targeted by different gears. A potentially exciting research area, which was beyond the scope of this paper, would be to examine how gear sensitivity has changed over time.

Of course, we were still missing species-specific data for many of the most commonly captured species, so we expect that these figures might change when critical data gaps are filled. Our analysis helped to highlight critical research priorities for how species important to the fishery respond to coral loss. In particular, there were five species [***Leptoscarus vaigiensis***, Marbled or green parrotfish; ***Lethrinus lentjan***, Pink ear emperor (genus level average exists); ***Calotomus carolinus***, Carolines parrotfish; ***Cheilio inermis***, Cigar wrasse; and ***Anampses caeruleopunctatus***, Bluespotted wrasse (genus level average exists)] that accounted for ~15-30% of the catch per gear (Table S4). By collecting data on these five species, we would have species-specific responses for >72-88% of the catch abundance for each gear (Table S4). Critically several of these species are not coral associated, such as Leptoscarus vaigiensis, which is predominantly found in sea grass habitat. Sea grass habitats can be severely affected by temperature anomalies, sea level rise, and changes to rainfall patterns [[e.g. 17](#_ENREF_17)], all of which are expected to change under a climate change scenario. However, we do not yet have data on species-specific responses to changes in sea grass ecosystems, but hope our framework and data gaps will enable this type of research to be collected and compiled, as has been done with coral reefs.

Table S4. Missing information on five species creates a significant gap in our understanding on how species respond to coral mortality. Column 1 shows the relative abundance of the five critical species without species-specific data on responses to coral mortality by gear type. Column 2 shows existing species level data by gear type. Column 3 shows the proportion of catch data that we would have species-specific understandings of if just five species were studied.

|  | 1. Relative abundance of 5 species | 2. Species-specific data relative abundance | 1+2 |
| --- | --- | --- | --- |
| Beach seine | 28.5 | 49.9 | 78.4 |
| Line | 21.2 | 52.8 | 73.9 |
| Net | 16.1 | 55.6 | 71.7 |
| Spear | 30.0 | 48.2 | 78.2 |
| Trap | 19.2 | 69.2 | 88.5 |

**Adaptive Capacity**

The occupational mobility measure (recorded as whether the respondent had changed jobs in the last 5 years AND preferred the current occupation) was extremely low (highest was <8% of respondents in Gazi). This was surprising because a previous study [[16](#_ENREF_16)] found that in the broader community (i.e. not only surveying fishermen and resource users) 16-34% of respondents had changed to a preferred occupation. In Mayungu, our 2012 survey of resource users found 0% of respondents had occupational mobility according to this indicator, whereas 34% of broader community members did in a 2005 survey [[16](#_ENREF_16)]. Our findings suggest that few people had recently (past 5 years) transitioned into fisheries and preferred it to their previous occupation. Because of the low variability in this particular variable, and the high proportion of communities with 0s, we decided to drop it from our index.

Because this study sought to investigate methods for assessing vulnerability, we compared adaptive capacity metrics that were both weighted (i.e. where each indicator will contribute differently to the overall score) and unweighted (where each indicator will contribute evenly). We weighted the indicators using a Principal Component Analysis (PCA), which is an ordination technique that is often used by social scientists to construct indices [[e.g. 18](#_ENREF_18)]. Our first analytical steps were to examine whether there were high levels of correlations among our adaptive capacity measures, and we found that several of the variables were significantly correlated (Table S5). Absence of debt was, significantly negatively correlated with Access to Credit (rho=-0.976, p<0.01), meaning that those who were in debt, unsurprisingly also reported that they had access to credit. Given the correlation and that they reflect the same process we removed debt from our analysis.

To calculate weights for our indicators based on the Principal Component Analysis, we used the eigenvalues of each principal component (Table S9) and the absolute (i.e. positive) values of the factors loadings on PC1, PC2 and PC3 (Table S10, Table S11). We consider those absolute factor loadings as representing capacity of each indicator to explain different dimensions (whether positive or negative). Then we calculated the average of each normalized indicator per community, and use those to calculate the unweighted average and weighted average of those indicators, which is our adaptive capacity.

The weights of each indicator is calculated as follows:


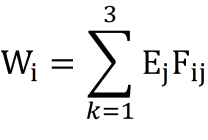


Where: i = indicator, k = principal component, W_i_, =Weight of indicator (Table S11) I, E_j_, =Eigenvalue of principal component k (Table S9 and Table S11), and F_ij_, = Factor loading of indicator i on principal component k (Table S10 and Table S11). We then normalized the weights of all indicators, so that the sum of all weights (9 indicators) is equal to 1 (Table S11).

Those weights are then used to calculate the weighted adaptive capacity index for each community as follows:


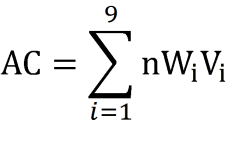


Where: i = indicator, nW_i_, normalized weight of indicator I, V_i_, and normalized value of indicator i

Table S5 and Fig. 6 (main text) show that some adaptive capacity indicators were correlated. Using weighted averages aims to reflect the relative importance of different adaptive capacity indicators but has little influence on the final adaptive capacity scores. Given the current, emerging theory on adaptive capacity, we did not feel there were well-justified rationales for weighting indicators so a straightforward average of normalized values was used. However final adaptive capacity scores may be sensitive to weightings, particularly for example the weight placed on occupational multiplicity. If this indicator was heavily weighted as important for adaptive capacity, then poorer, less developed communities that had high occupational multiplicity, would be assessed as having higher adaptive capacity. Conversely, adaptive capacity indicator weightings that prioritized the importance of wealth and infrastructure would penalize the adaptive capacity score of these communities.

This exercise of constructing each component of adaptive capacity by combining indicators highlighted the important of the normalization procedure. Careful thinking is needed to choose appropriate and meaningful bounding for each indicator, because each indicator has to capture the largest possible variation in the original variable. We cannot always simply consider the minimum and maximum values of the original variables as the 0 and 1 of the normalized indicator. For example, if a variable does not have much variation with the communities we consider (but we know it can have more variation), or if a variable has outliers, then the minimum and maximum will nott be meaningful as bounding and using them would reduce or amplify the variation in the normalized indicator and so its relative importance to other indicators when combining them. So it is important to go through each indicator and think carefully about its bounding for normalization and what it means, to have appropriate indicators.

References

1. McClanahan T, Kaunda-Arara B (1996) Fishery recovery in a coral reef marine park and its effect on the adjacent fishery. Conservation Biology 10: 1187-1199.

2. Allen GR, Werner TB (2002) Coral reef fish assessment in the 'coral triangle' of southeastern Asia. Environmental Biology of Fishes 65: 209-214.

3. Maina J, Venus V, McClanahan TR, Ateweberhan M (2008) Modelling susceptibility of coral reefs to environmental stress using remote sensing data and GIS models in the western Indian Ocean. Ecological Modelling 212: 180-199.

4. Maina J, McClanahan TR, Venus V, Ateweberhan M, Madin J (2011) Global gradients of coral exposure to environmental stresses and implications for local management. PLoS ONE 6: e23064.

5. ReefBase (2008) Knowledgebase for Lessons Learned and Best Practices in te Management of Coral Reefs. REEFBASE.

6. McClanahan TR, Ateweberhan M, Graham NAJ, Wilson SK, Ruiz Sebasti√°n C, et al. (2007) Western Indian Ocean coral communities: Bleaching responses and susceptibility to extinction. Marine Ecology Progress Series 337: 1-13.

7. McClanahan TR, Maina J, Moothien Pillay R, Baker AC (2005) Effects of geography, taxa, water flow, and temperature variation on coral bleaching intensity in Mauritius. Marine Ecology Progress Series 298: 131-142.

8. Graham NAJ, Chabanet P, Evans RD, Jennings S, Letourneur Y, et al. (2011) Extinction vulnerability of coral reef fishes. Ecology Letters 14: 341-348.

9. McClanahan TR (1992) Resource utilization, competition and predation: a model and example from coral reef grazers. Ecological Modelling 61: 195-215.

10. McClanahan T (1995) A coral reef ecosystem-fisheries model - impacts of fishing intensity and catch selection on reef structure and processes. Ecological Modeling 80: 1-19.

11. Cinner J, Bodin O (2010) Livelihood diversification in tropical coastal communities: a network-based approach to analyzing 'livelihood landscapes'. PLoS ONE 5: e11999.

12. Pratchett MS, Hoey AS, Wilson SK, Messmer V, Graham NAJ (2011) Changes in biodiversity and functioning of reef fish assemblages following coral bleaching and coral loss. Diversity 3: 424-452.

13. Cinner JE, McClanahan TR, Graham NAJ, Pratchett MS, Wilson SK, et al. (2009) Gear-based fisheries management as a potential adaptive response to climate change and coral mortality. Journal of Applied Ecology 46: 724-732.

14. McClanahan TR, Hicks CC (2011) Changes in life history and ecological characteristics of coral reef fish catch composition with increasing fishery management. Fisheries Management and Ecology 18: 50-60.

15. Randall JE, Allen GR, Steene R, editors (1997) Fishes of the Great Barrier Reef and Coral Sea. 2nd ed. Hawaii: University of Hawaii Press.

16. McClanahan TR, Cinner JE, Maina J, Graham NAJ, Daw TM, et al. (2008) Conservation action in a changing climate. Conservation Letters 1: 53-59.

17. Rasheed MA, Unsworth RKF (2011) Long-term climate-associated dynamics of a tropical seagrass meadow: implications for the future. Marine Ecology Progress Series 422: 93-103.

18. Pollnac R, Crawford B (2000) Assessing behavioural aspects of coastal resource use. Narragansett, Rhode Island, USA: Coastal Resources Center, University of Rhode Island. 139 p.

Table S5. Spearman correlations between the 11 adaptive capacity indicators (correlations conducted at the community scale). ** significant at 0.01, * significant at 0.05

|  | Access to Credit | No Debt | Human Agency | Occupational Multiplicity | Capacity to Change | Trust | Gear Diversity | Social Capital | Occupational Mobility | Community Infrastructure | MSL |
| --- | --- | --- | --- | --- | --- | --- | --- | --- | --- | --- | --- |
| Access to Credit | 1.000 |  |  |  |  |  |  |  |  |  |  |
| No Debt | **-.976**** | 1.000 |  |  |  |  |  |  |  |  |  |
| Human Agency | -.158 | .091 | 1.000 |  |  |  |  |  |  |  |  |
| Occupational Multiplicity | .321 | -.358 | .419 | 1.000 |  |  |  |  |  |  |  |
| Capacity to Change | **.709*** | **-.636*** | .158 | .079 | 1.000 |  |  |  |  |  |  |
| Trust | -.297 | .152 | -.249 | -.382 | -.418 | 1.000 |  |  |  |  |  |
| Gear Diversity | .505 | -.505 | .378 | .219 | .626 | -.523 | 1.000 |  |  |  |  |
| Social Capital | **.758*** | **-.758*** | .116 | .139 | **.661*** | -.224 | .620 | 1.000 |  |  |  |
| Occupational Mobility | -.143 | .164 | -.216 | -.471 | -.075 | .314 | -.541 | -.157 | 1.000 |  |  |
| Community Infrastructure | .297 | -.321 | -.207 | -.479 | .297 | .297 | .182 | **.709*** | .157 | 1.000 |  |
| MSL | .055 | -.079 | .286 | -.418 | .224 | .042 | .164 | .285 | .068 | .467 | 1.000 |

Table S6. Ecological vulnerability indicators of exposure, sensitivity and recovery potential for 15 ecological sites. Detailed description of the rational for indicators and how indicators were calculated can be found in Table 1 and the Methods.

|  |  |  |  | *Exposure* | *Sensitivity* | | *Recovery potential* | | | | | | |
| --- | --- | --- | --- | --- | --- | --- | --- | --- | --- | --- | --- | --- | --- |
| **Site no.** | **Community** | **Management** | **Ecological site** | **Exposure, stress model** | **Coral susceptibility index** | **Fish susceptibility index** | **Coral cover, %** | **Coral size, CV** | **Coral richness, no. genera** | **Rugosity** | **Fish biomass, kg ha^-1^** | **Herbivore diversity, Simpson index** | **Grazing to Algal production, kg day^-1^** |
| 1 | Vanga | Fished | Vanga | 0.65 | 13.34 | 0.43 | 20.96 | 0.47 | 19 | 1.22 | 229.72 | 0.14 | 27.82 |
| 2 | Shimoni-fished | Fished | Changai | 0.55 | 15.65 | 0.49 | 43.94 | 0.49 | 25 | 1.18 | 254.83 | 0.21 | 53.21 |
| 3 | Shimoni-park | Park | Kisite | 0.60 | 17.19 | 0.39 | 49.91 | 0.63 | 25 | 1.30 | 1643.39 | 0.59 | 58.84 |
| 4 | Funzi | Fished | Funzi | 0.59 | 15.56 | NA | 30.63 | NA | 20 | NA | NA | NA | NA |
| 5 | Gazi | Fished | Gazi | 0.60 | 15.40 | 0.31 | 12.02 | 0.59 | 18 | 1.33 | 107.76 | 0.04 | -21.70 |
| 6 | Bamburi-fished | Fished | RasIwatine | 0.67 | 16.04 | 0.30 | 7.10 | 0.39 | 15 | 1.22 | 96.46 | 0.08 | -71.96 |
| 7 | Bamburi-park | Park | Mombasa | 0.67 | 13.74 | 0.35 | 20.23 | 0.71 | 19 | 1.27 | 867.97 | 0.72 | -37.95 |
| 8 | Mtwapa | Fished | Mtwapa | 0.59 | 15.82 | 0.33 | 26.36 | 0.61 | 22 | 1.23 | 153.25 | 0.20 | -43.42 |
| 9 | Kanamai-fished | Fished | Kanamai | 0.59 | 17.90 | 0.34 | 34.77 | 0.36 | 14 | 1.21 | 70.60 | 0.02 | 34.01 |
| 10 | Kanamai-tengefu | Tengefu | Mradi | 0.59 | 15.51 | 0.37 | 54.58 | 0.61 | 22 | 1.27 | 440.43 | 0.52 | 18.56 |
| 11 | Kuruwitu-tengefu | Tengefu | Kuruwitu | 0.63 | 16.98 | 0.34 | 26.16 | 0.54 | 13 | 1.21 | 364.08 | 0.55 | -12.66 |
| 12 | Takaungu | Fished | Takaungu | 0.63 | 17.28 | 0.36 | 0.76 | 0.59 | 14 | 1.18 | 91.10 | 0.55 | -165.38 |
| 13 | Mayungu-park | Park | Watamu | 0.62 | 14.22 | 0.34 | 31.51 | 0.78 | 16 | 1.55 | 1320.94 | 0.54 | -35.42 |
| 14 | Mayungu-fished | Fished | Mayungu | 0.62 | 16.33 | 0.34 | 7.28 | 0.42 | 17 | 1.17 | 204.99 | 0.43 | -151.95 |
| 15 | Mayungu-park | Park | Malindi | 0.68 | 17.63 | 0.37 | 27.18 | 0.48 | 21 | 1.36 | 711.05 | 0.26 | -65.08 |

Table S7. Dimensions of ecological vulnerability for 17 coral reef sites in Kenya. Ecological vulnerability was calculated from normalized and weighted indicators as (Exposure + Sensitivity) – Recovery Potential. Sites are ranked from most vulnerable to least vulnerable.

| **Site no.** | **Community** | **Management** | **Ecological vulnerability** | **Exposure** | **Sensitivity** | **Recovery potential** |
| --- | --- | --- | --- | --- | --- | --- |
| 1 | Vanga | Fished | 0.65 | 0.65 | 0.18 | 0.17 |
| 2 | Shimoni | Fished | 0.67 | 0.55 | 0.35 | 0.23 |
| 3 | Shimoni | Park | 0.51 | 0.6 | 0.3 | 0.4 |
| 4 | Funzi | Fished | 0.74 | 0.59 | 0.34 | 0.2 |
| 5 | Gazi | Fished | 0.59 | 0.6 | 0.14 | 0.15 |
| 6 | Bamburi | Fished | 0.74 | 0.67 | 0.15 | 0.08 |
| 7 | Bamburi | Park | 0.51 | 0.67 | 0.11 | 0.28 |
| 8 | Mtwapa | Fished | 0.59 | 0.59 | 0.18 | 0.17 |
| 9 | Kanamai | Fished | 0.73 | 0.59 | 0.28 | 0.14 |
| 10 | Kanamai | Tengefu | 0.51 | 0.59 | 0.21 | 0.29 |
| 11 | Kuruwitu | Tengefu | 0.67 | 0.63 | 0.24 | 0.2 |
| 12 | Takaungu | Fished | 0.79 | 0.63 | 0.27 | 0.11 |
| 13 | Mayungu (Watamu) | Park | 0.42 | 0.62 | 0.12 | 0.31 |
| 14 | Mayungu | Fished | 0.73 | 0.62 | 0.21 | 0.1 |
| 15 | Mayungu (Malindi) | Park | 0.76 | 0.68 | 0.3 | 0.22 |

Table S8. The 11 adaptive capacity indicators aggregate values at community level shown as % or mean ± standard deviations

| **Community** | **Access Credit** | **No Debt** | **Human Agency** | **Occupational Multiplicity** | **Capacity to Change** | **Trust** | **Gear Diversity** | **Social Capital** | **Occupational Mobility** | **Community Infrastructure** | **MSL** |
| --- | --- | --- | --- | --- | --- | --- | --- | --- | --- | --- | --- |
| Bamburi | 43.3 | 80.0 | 63.3 | 1.90 (±1.32) | 73.3 | 3.07 (±1.05) | 1.23 (±0.53) | 1.30 (±0.75) | 3.3 | 24 | 0.7 (±1.23) |
| Funzi | 15.0 | 90.0 | 90.0 | 2.30 (±1.49) | 60.0 | 3.26 (±0.9) | 1.33 (±0.49) | 0.30 (±0.66) | 0.0 | 09 | -0.25 (±0.32) |
| Gazi | 26.3 | 89.5 | 47.4 | 1.95 (±0.9) | 57.9 | 3.54 (±0.81) | 1.06 (±0.25) | 0.53 (±0.6) | 7.9 | 13 | -0.24 (±0.88) |
| Kanamai | 60.7 | 64.3 | 53.6 | 2.25 (±1.53) | 89.3 | 3.04 (±0.87) | 1.35 (±0.49) | 0.89 (±0.5) | 3.6 | 10 | -0.32 (±0.61) |
| Kuruwitu | 58.8 | 70.6 | 70.6 | 2.41 (±0.78) | 82.4 | 3.06 (±0.99) | 1.48 (±0.58) | 1.76 (±0.74) | 0.0 | 14 | -0.34 (±0.63) |
| Mayungu | 53.3 | 73.3 | 46.7 | 2.83 (±3.34) | 53.3 | 3.48 (±0.71) | 1.13 (±0.34) | 0.80 (±0.89) | 0.0 | 12 | -0.2 (±0.97) |
| Mtwapa | 43.8 | 75.0 | 46.9 | 1.97 (±1.75) | 65.6 | 3.27 (±0.85) | 1.48 (±0.58) | 0.97 (±0.47) | 0.0 | 19 | 0.08 (±1.12) |
| Shimoni | 60.0 | 55.0 | 70.0 | 2.53 (±2.39) | 82.5 | 3.28 (±0.65) | 1.58 (±0.78) | 1.38 (±0.81) | 0.0 | 18 | 0.35 (±1.18) |
| Takaungu | 33.3 | 81.5 | 66.7 | 3.00 (±2.39) | 22.2 | 3.04 (±0.85) | 1.25 (±0.44) | 0.74 (±0.53) | 0.0 | 10 | -0.28 (±0.74) |
| Vanga | 40.7 | 77.8 | 66.7 | 1.81 (±0.92) | 48.1 | 3.54 (±0.83) | 1.15 (±0.36) | 0.85 (±0.53) | 50 | 16 | 0.39 (±1.17) |

Table S9. Eigenvalues and percentage of variation explained by the different PCs

|  | Eigenvalues | % of Variance | Cumulative % |
| --- | --- | --- | --- |
| PC1 | 0.082 | 41.75 | 41.75 |
| PC2 | 0.049 | 24.71 | 66.47 |
| PC3 | 0.031 | 15.79 | 82.26 |

**Table S10. Factor loadings of adaptive capacity indicators. Factor loadings above 0.4 (in bold) on any given Principal Component are generally considered to contribute substantially to that Component.**

|  | PC1 | PC2 | PC3 |
| --- | --- | --- | --- |
| Social Capital | **0.842** | 0.182 | -0.045 |
| Capacity to Change | **0.813** | 0.319 | 0.059 |
| Access Credit | **0.731** | **0.410** | -0.331 |
| Community Infrastructure | **0.697** | **-0.641** | -0.209 |
| Gear Diversity | **0.529** | **0.435** | **0.473** |
| Trust | -0.346 | -0.336 | -0.335 |
| Occupational Multiplicity | 0.004 | **0.767** | 0.292 |
| MSL | **0.491** | **-0.757** | 0.342 |
| Human Agency | -0.027 | -0.072 | **0.971** |

**Table S11. Absolute factor loadings, weights and normalised weights of each adaptive capacity indicator.**

|  | PC1 | PC2 | PC3 | Weight | Normalized Weight |
| --- | --- | --- | --- | --- | --- |
| **Eigenvalues** | **0.082** | **0.049** | **0.031** |  |  |
| Social Capital | 0.842 | 0.182 | 0.045 | 0.079 | 0.122 |
| Capacity to Change | 0.813 | 0.319 | 0.059 | 0.084 | 0.129 |
| Access Credit | 0.731 | 0.410 | 0.331 | 0.090 | 0.138 |
| Community Infrastructure | 0.697 | 0.641 | 0.209 | 0.095 | 0.145 |
| Gear Diversity | 0.529 | 0.435 | 0.473 | 0.079 | 0.121 |
| Trust | 0.346 | 0.336 | 0.335 | 0.055 | 0.084 |
| Occupational Multiplicity | 0.004 | 0.767 | 0.292 | 0.047 | 0.071 |
| MSL | 0.491 | 0.757 | 0.342 | 0.088 | 0.134 |
| Human Agency | 0.027 | 0.072 | 0.971 | 0.036 | 0.055 |


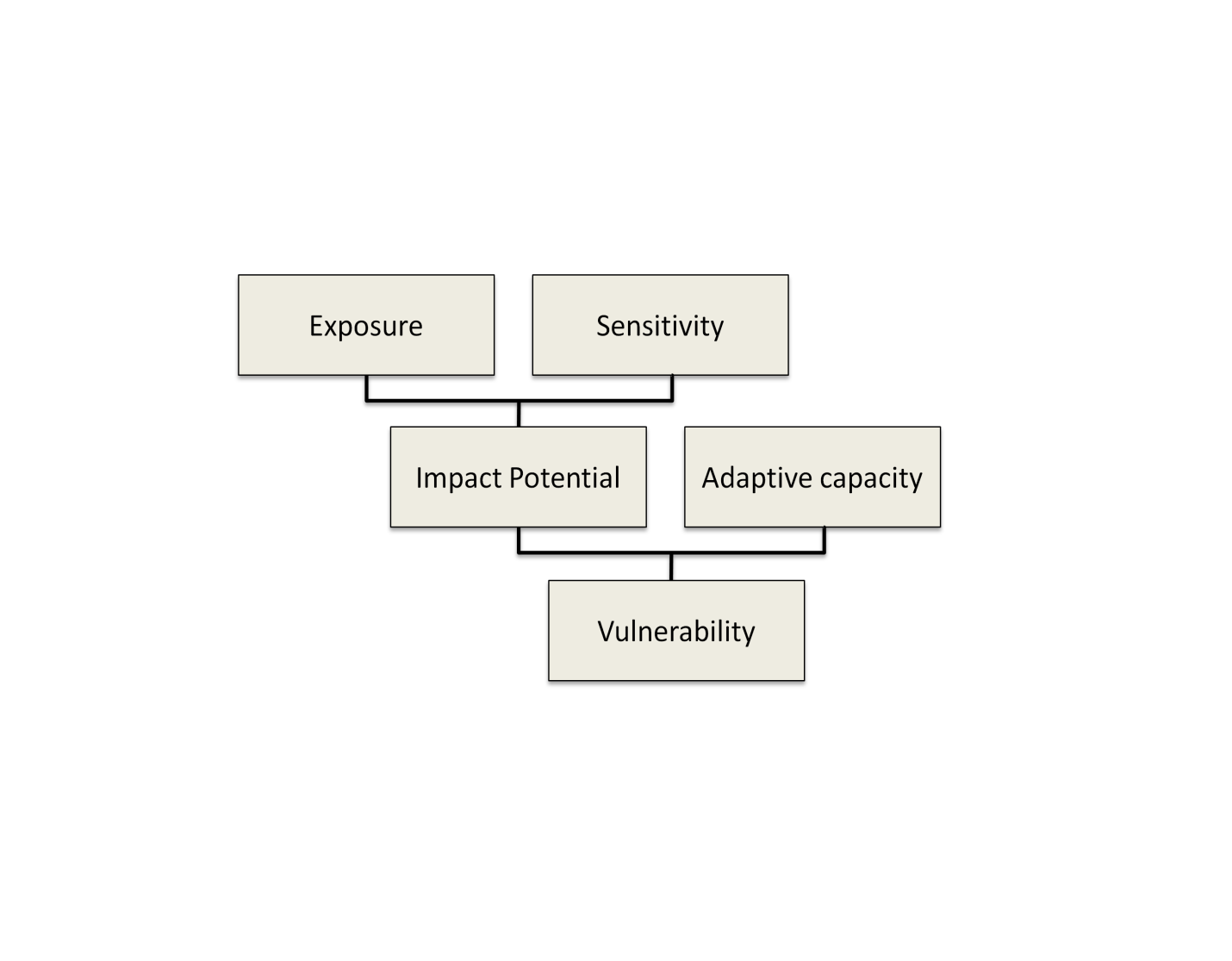


**Figure S7. Intergovernmental Panel on Climate Change (IPCC) conceptual framework of vulnerability to climate change.**
